# *SpatialWavePredict*: a tutorial-based primer and toolbox for forecasting growth trajectories using the ensemble spatial wave sub-epidemic modeling framework

**DOI:** 10.1186/s12874-024-02241-2

**Published:** 2024-06-07

**Authors:** Gerardo Chowell, Amna Tariq, Sushma Dahal, Amanda Bleichrodt, Ruiyan Luo, James M. Hyman

**Affiliations:** 1 Department of Population Health Sciences, School of Public Health, Georgia State University, Atlanta, GA USA; 2 Department of Applied Mathematics, Kyung Hee University, Yongin, 17104 Korea; 3 Department of Pediatrics, School of Medicine, Stanford University, Palo Alto, CA USA; 4 Department of Mathematics, Tulane University, New Orleans, LA USA

**Keywords:** MATLAB toolbox, Real-time forecasting, Dynamic growth model, Spatial wave sub-epidemic wave model, Ensemble model, Complex epidemic patterns

## Abstract

**Background:**

Dynamical mathematical models defined by a system of differential equations are typically not easily accessible to non-experts. However, forecasts based on these types of models can help gain insights into the mechanisms driving the process and may outcompete simpler phenomenological growth models. Here we introduce a friendly toolbox, *SpatialWavePredict*, to characterize and forecast the spatial wave sub-epidemic model, which captures diverse wave dynamics by aggregating multiple asynchronous growth processes and has outperformed simpler phenomenological growth models in short-term forecasts of various infectious diseases outbreaks including SARS, Ebola, and the early waves of the COVID-19 pandemic in the US.

**Results:**

This tutorial-based primer introduces and illustrates a user-friendly MATLAB toolbox for fitting and forecasting time-series trajectories using an ensemble spatial wave sub-epidemic model based on ordinary differential equations. Scientists, policymakers, and students can use the toolbox to conduct real-time short-term forecasts. The five-parameter epidemic wave model in the toolbox aggregates linked overlapping sub-epidemics and captures a rich spectrum of epidemic wave dynamics, including oscillatory wave behavior and plateaus. An ensemble strategy aims to improve forecasting performance by combining the resulting top-ranked models. The toolbox provides a tutorial for forecasting time-series trajectories, including the full uncertainty distribution derived through parametric bootstrapping, which is needed to construct prediction intervals and evaluate their accuracy. Functions are available to assess forecasting performance, estimation methods, error structures in the data, and forecasting horizons. The toolbox also includes functions to quantify forecasting performance using metrics that evaluate point and distributional forecasts, including the weighted interval score.

**Conclusions:**

We have developed the first comprehensive toolbox to characterize and forecast time-series data using an ensemble spatial wave sub-epidemic wave model. As an epidemic situation or contagion occurs, the tools presented in this tutorial can facilitate policymakers to guide the implementation of containment strategies and assess the impact of control interventions. We demonstrate the functionality of the toolbox with examples, including a tutorial video, and is illustrated using daily data on the COVID-19 pandemic in the USA.

**Supplementary Information:**

The online version contains supplementary material available at 10.1186/s12874-024-02241-2.

## Background

 Developing reliable methods for forecasting dynamic growth processes is critical for decision-making in problems ranging from predicting the weather, forecasting the trajectory of an emerging epidemic, the growth or decline of economic variables, election outcomes, and sporting events [1]. While statistical methods such as ARIMA and exponential smoothing are robust and broadly competitive for forecasting time series [2–6], dynamical mathematical models defined by a system of differential equations are typically not easily accessible to non-experts. However, forecasts based on these types of models can help characterize the mechanisms driving the process [7]. They may offer higher forecasting performance than purely statistical approaches based on statistical evaluation criteria like mean absolute and squared errors [8–11]. Here we focus on dynamical models that can characterize growth processes that give rise to waves of variable shapes and sizes [12–14]. The complexity of this family of growth models ranges from single differential equation models with a few parameters, such as the 3-parameter generalized-logistic growth model (GLM) [14], to systems of ordinary differential equations (ODEs) that capture diverse wave dynamics by aggregating multiple asynchronous growth processes [13]. The spatial wave sub-epidemic framework has outperformed simpler phenomenological growth models in forecasts of various infectious diseases, including severe acute respiratory syndrome (SARS), Ebola, and the early waves of the coronavirus disease 2019 (COVID-19) pandemic in the United States (US) [13, 15].

This tutorial paper introduces a user-friendly MATLAB toolbox to fit and forecast time-series trajectories using the spatial wave sub-epidemic dynamic growth model based on ordinary differential equations, which was initially developed to characterize and derive short-term forecasts of epidemic trajectories [13, 16]. This mathematical framework characterizes time-series trajectories by aggregating multiple asynchronous growth processes. Each growth process (i.e., sub-epidemic) is modeled using a simple phenomenological growth model such as the generalized logistic growth model (GLM). This framework supports a family of growth models that yield similar fits to the calibration data, but their corresponding forecasts could produce diverse trajectories. Hence, we also incorporate ensemble techniques to combine the resulting models to boost forecasting performance [16, 17].

This toolbox is written for a diverse audience, including students training in time-series forecasting. It allows the user to conduct parameter estimation and forecasting with quantified uncertainty and evaluate forecasting performance using a set of standard metrics, including the coverage of the 95% prediction interval and the weighted interval score, which account for the uncertainty of the predictions. The toolbox allows scientists and policymakers to generate short-term forecasts by relying on minimal data of the process of interest, such as an unfolding epidemic or natural disaster.

The toolbox provides prediction intervals and allows the user to employ different estimation methods, assumptions of the error structure, and forecasting horizons. For instance, the toolbox includes estimation methods such as the nonlinear least squares estimation and maximum likelihood estimation (MLE) with different assumptions about the error structure of the observed data, including Poisson, negative binomial, and normal distributions, as well as quantification of the uncertainty based on a parametric bootstrapping approach. The model also provides flexibility to choose the underlying building block of the growth process. In addition, the toolbox includes functions to derive weighted and unweighted ensembles based on the resulting top-ranked models. The full functionality of the toolbox is illustrated using daily time series of COVID-19 cases in the US, and in the process, shows that this framework outcompetes simpler single growth models and simple time-series models (e.g., ARIMA, GAM, SLR) in calibration and forecasting performance.

We start by describing the format of the input time-series data, followed by the methods employed for parameter estimation. Next, we describe the underlying methodology, user parameters, and functions to calibrate, evaluate, and display the model fits. Finally, we introduce the functions to generate, display, and quantify the performance of model-based forecasts with specific examples in the context of the daily COVID-19 case data reported in the USA. A tutorial video that demonstrates the toolbox functionality is available at: https://www.youtube.com/watch?v=qxuF_tTzcR8&t=47s.

## Implementation

In this section, we describe the methods implemented in this toolbox and provide a brief overview of the toolbox functions.

### Installing the toolbox

- Download the MATLAB code located in the folder ***spatialWave_subepidemicFramework code*** from the GitHub repository: https://github.com/gchowell/spatial_wave_subepidemic_framework.
- Create an ‘input’ folder in your working directory where your input data will be stored.
- Create an ‘output’ folder in your working directory where the output files will be stored.
- Open a MATLAB session.

### Overview of the toolbox functions

The methodological workflow of the tutorial is organized as follows: (1) plotting model simulations, (2) fitting the models to data with quantified uncertainty, (3) plotting the resulting model fits and calibration performance metrics, and (4) plotting model-based forecasts and the associated forecasting performance metrics. Table 1 and Supplementary Table 1 list the names of both user and internal functions associated with the toolbox, along with a brief description of their role. As described below, the user needs to specify the parameters related to model fitting and forecasting in the default options_fit.m and options_forecast.m files.

**Table 1:** Description of the user functions available in the *SpatialWavePredict* toolbox

Function	Role
options.m	Specifies the parameters related to model fitting, including the characteristics of the time series data, the sub-epidemic model, parameter estimation method, error structure, smoothing, and calibration period. The structure of the options.m file is given in **Supplementary Text ** 1.
options_forecast.m	Specifies the parameters related to the forecast, including the forecasting period, the type of ensemble weight for the ensemble models, and whether the forecasts will be evaluated. The structure of the options_forecast.m file is given in **Supplementary Text ** 2.
plot_SW_subepidemic.m	Plots simulations of the spatial wave sub-epidemic model.
Run_SW_subepidemicFramework.m	Derives the top-ranking sub-epidemic wave models to data with quantified uncertainty.
plotRankings_SW_subepidemicFramework.m	Plots the mean model fits of the top-ranking models, including their sub-epidemic profiles, and the associated quality of model fit metrics, including the AICc, the relative likelihood, and the evidence ratio.
plotFit_SW_subepidemicFramework.m	Displays the model fit and 95% prediction interval, as well as the empirical distribution of the parameters. It also saves output .csv files in the output folder with the model fit, the parameter estimates, including 95% CIs, and the calibration performance metrics.
plotForecast_SW_subepidemicFramework.m	Displays the model-based forecast and the performance metrics of the forecast. Moreover, the data associated with the forecasts, the parameter estimates, as well as the calibration and forecasting performance metrics are saved as .csv files in the output folder.

### Parameter estimation method

Let $f\left(t,{\Theta }\right)$ denote the expected curve of the epidemic’s trajectory. We can estimate model parameters ${\Theta }$ by fitting the model solution to the observed data via nonlinear least squares [18] or maximum likelihood estimation with specific assumptions about the error structure in the data [19] by specifying parameter <method1> in the options.m file. For nonlinear least squares (i.e., <method1> =0), this is achieved by searching for the set of parameters $\widehat{\varTheta }$ that minimizes the sum of squared differences between the smoothed data ${y}_{{t}_{j}=}{y}_{{t}_{1}, }{y}_{{t}_{2}}\dots .{y}_{{t}_{{n}_{d}}}$ and the model mean, corresponding to $f(t,{\Theta })$. That is, ${\Theta }=\left({C}_{thr},r,p,q,{K}_{0}\right)$ in the sub-epidemic wave model (given below) is estimated by $\widehat{{\Theta }}=\text{arg}\text{min} \sum\nolimits _{j=1}^{{n}_{d}}{(f\left({t}_{j},{\Theta }\right)-{y}_{{t}_{j}})}^{2}$. We estimate the parameter ${C}_{thr}$ through simple discretization of its range of plausible values. Our estimation procedure consists of two steps. First, for each ${C}_{thr}$, we search for the set of parameters $(r,p,q,{K}_{0})$ that yield the best fit to the data. Then we choose ${C}_{thr}$and the corresponding estimates of other parameters leading to the overall best-fit to the data.

Nonlinear least squares estimation weighs each of the data points equally and does not explicitly require a specific distributional assumption for ${y}_{t}$, except for the first moment $E\left[{y}_{t}\right]=f({t}_{i};\varTheta )$. That is, the mean of the observed data at time *t* is equivalent to the expected count denoted by $f\left(t,{\Theta }\right)$at time *t* [20]. This method yields asymptotically unbiased point estimates regardless of any misspecification of the variance-covariance error structure. Hence, the estimated model mean $f({t}_{i},\widehat{{\Theta }})$ yields the best fit to observed data ${y}_{{t}_{i}}$in terms of squared L2 norm. We can solve the nonlinear least squares optimization problem using the *fmincon* function in MATLAB. Moreover, we also employ MATLAB’s MultiStart feature to specify the number of random initial guesses of the model parameters using the parameter <numstartpoints> in the options.m file in order to search thoroughly for a global minimum, check that the solution is unique, and the parameters are identifiable.

We can also estimate parameters via maximum likelihood estimation (MLE) [19] and assume different error structures in the data (e.g., Poisson, negative binomial). The log-likelihood expressions derived for different error structures are specified below.

#### Poisson

For a Poisson error structure, the full log-likelihood of Poisson (i.e., <method1> =1) is given by:

$$\sum\limits _{i=1}^{n}\left\{{y}_{i}ln\left({\mu }_{i}\right)-ln({y}_{i}!)-{\mu }_{i}\right\},$$

where ${\mu }_{i}=f ({t}_{i},\theta )$ denotes the mean of ${y}_{i}$at time ${t}_{i}$. The number of parameters is just the number of parameters estimated in the dynamical model based on ordinary differential equations.

#### Negative binomial

Let $r>0$ denote the number of failures until the experiment is stopped, $p\in [0, 1]$ denote the success probability in each experiment. The number of successes *y* before the r-th failure occurs has a **negative binomial distribution** given by:

$$f\left(y|r,p\right)=\left(\genfrac{}{}{0pt}{}{r+y-1}{y}\right){p}^{y }{(1-p)}^{r}=\frac{1}{y!}\prod\nolimits_{j=0}^{y-1}\left(j+r\right) .{p}^{y}({1-p)}^{r}$$

with $\text{m}\text{e}\text{a}\text{n}=\mu =\frac{rp}{(1-p)}$, variance $={\sigma }^{2}=\frac{rp}{{\left(1-p\right)}^{2}}> \mu$. For *n* observations ${y}_{1}, \dots ,{y}_{n}$, the full log-likelihood is

1.1 $$l(r,p)={\sum }_{i=1}^{n}\left\{\left\{{\sum }_{j=0}^{{y}_{i}-1}\text{l}\text{n}(j+r)\right\}+ {y}_{i}\text{ln}\left({p}_{i}\right)+rln\left(1-{p}_{i}\right)-\text{l}\text{n}({y}_{i}!)\right\},$$

which can be expressed with $\mu$ and *σ* ^2^ by plugging-in $p=1-\frac{{\upmu }}{{{\upsigma }}^{2}}$ and $r=\frac{{\mu }^{2}}{{\sigma }^{2}-\mu }$.

There are different types of variances commonly used in a negative binomial distribution. If the variance scales linearly with the mean: ${\sigma }^{2}=\mu +\alpha \mu$, (i.e., <method1> = 3 in options.m), then $p=\frac{\alpha }{1+\alpha }$ and $r=\mu /\alpha$. Let $\mu =f \left(t,\theta \right)$be the mean curve to be estimated from the differential equation. The full log-likelihood (1.1) can be expressed as follows:

1.2 $$l\left(\theta ,\alpha \right)=\sum\nolimits _{i=1}^{n}\left\{\left\{\sum\nolimits _{j=0}^{{y}_{i}-1}ln(j+{\alpha }^{-1}f({t}_{i},\theta \left)\right)\right\} +{y}_{i}ln\left(\alpha \right)-({y}_{i}+{\alpha }^{-1}f({t}_{i},\theta \left)\right)ln(1+\alpha )-ln({y}_{i}!)\right\}.$$

If the variance scales quadratically with the mean, ${\sigma }^{2}=\mu +\alpha {\mu }^{2}$ (i.e., <method1> =4 in options.m), then $p=\frac{\alpha \mu }{1+\alpha \mu }$and $r=1/\alpha$. The full log-likelihood (1.1) can be expressed as follows:

1.3 $$l\left(\theta ,\alpha \right)=\sum\nolimits _{i=1}^{n}\left\{\left\{\sum\nolimits _{j=0}^{{y}_{i}-1}ln(j+{\alpha }^{-1})\right\} +{y}_{i}ln\left(\alpha f\right({t}_{i},\theta \left)\right)-({y}_{i}+{\alpha }^{-1})ln(1+\alpha f({t}_{i},\theta \left)\right)-ln({y}_{i}!)\right\}.$$

The more general form of variance is ${\sigma }^{2}=\mu +\alpha {\mu }^{d}$ (i.e., <method1> =5 in options.m) with any $-{\infty }<d<{\infty }$. Then the full log-likelihood (1.1) can be expressed as follows:

1.4 $$l\left(\theta ,\alpha \right)=\sum\nolimits _{i=1}^{n}\left[\left\{\sum\nolimits _{j=0}^{{y}_{i}-1}ln(j+{\alpha }^{-1}{{\mu }_{i}}^{2-d})\right\}+{y}_{i}ln\left(\alpha {{\mu }_{i}}^{d-1}\right)-({y}_{i}+{\alpha }^{-1}{{\mu }_{i}}^{2-d})ln(1+\alpha {{\mu }_{i}}^{d-1})-ln({y}_{i}!)\right],$$

where $${\mu }_{i}=f ({t}_{i},\theta ).$$

The number of parameters is 1 plus the number of parameters in the dynamical model based on ordinary differential equations (ODE) for (1.2) ~ (1.3), and 2 plus the number of parameters in the dynamical model for (1.4) if *d* is also estimated via MLE. Assuming Poisson or negative binomial error structures in the data, we can estimate parameters using MLE by specifying parameters in the options.m file, such as <method1 >=1 & <dist1>=1 for Poisson and <method1> & <dist1>=3, <method1>=4 & <dist1 >=4, and <method1>=5 & <dist1> = 5 for the different negative binomial error structures described above.

## Parametric bootstrapping

To quantify parameter uncertainty, we follow a parametric bootstrapping approach which allows the computation of standard errors and related statistics in the absence of closed-form formulas [21]. We generate *B* bootstrap samples from the best-fit model $f(t,\widehat{\varTheta})$, with an assumed error structure specified using parameter <dist1> in the options.m file to quantify the uncertainty of the parameter estimates and construct confidence intervals. Typically, the error structure in the data is modeled using a probability model such as the Poisson or negative binomial distribution. Using nonlinear least squares (< method1> = 0), besides a normally distributed error structure (<dist1> = 0), we can also assume a Poisson (<dist1> = 1) or a negative binomial distribution (<dist1> = 2) whereby the variance-to-mean ratio is empirically estimated from the time series. To estimate this constant ratio, we group a fixed number of observations (e.g., 7 observations for daily data into a bin across time), calculate the mean and variance for each bin, and then estimate a constant variance-to-mean ratio by calculating the average of the variance-to-mean ratios over these bins.

Using the best-fit model $f(t,\widehat{\varTheta })$, we generate *B*-times replicated simulated datasets of size ${n}_{d}$, where the observation at time ${t}_{j }$is sampled from the corresponding distribution specified by <dist1>. Next, we refit the model to each of the *B* simulated datasets to re-estimate the parameters using the same estimation method for the bootstrap sample as for the original data. This allows us to quantify the uncertainty of the estimate using that method. The new parameter estimates for each realization are denoted by ${\widehat{{\Theta }}}_{b}$, where $b=\text{1,2},\dots ,B$. Using the sets of re-estimated parameters ($\widehat{\Theta}_{b}$), it is possible to characterize the empirical distribution of each estimate, calculate the variance, and construct confidence intervals for each parameter. The resulting uncertainty around the model fit can similarly be obtained from $f\left(t,{\widehat{{\Theta }}}_{1}\right),$ $f\left(t,{\widehat{{\Theta }}}_{2}\right),\dots ,f(t,{\widehat{{\Theta }}}_{B})$. We characterize the uncertainty using 300 bootstrap realizations (i.e., parameter <B> = 300 in the options.m file).

### Model-based forecasts with quantified uncertainty

Forecasting the model $f\left(t,\widehat{{\Theta }}\right), h$ days ahead is based on the estimate $f(t+h,\widehat{\Theta})$. The uncertainty of the forecasted value can be obtained using the previously described parametric bootstrap method. Let

$$f\left(t+h,{\widehat{{\Theta }}}_{1}\right), f\left(t+h,{\widehat{{\Theta }}}_{2}\right),\dots ,f(t+h,{\widehat{{\Theta }}}_{B})$$

denote the forecasted value of the current state of the system propagated by a horizon of *h* time units, where ${\widehat{{\Theta }}}_{b}$ denotes the estimation of parameter set ${\Theta }$ from the *b*_*th*_ bootstrap sample. We can use these values to calculate the bootstrap variance to measure the uncertainty of the forecasts and use the 2.5% and 97.5% percentiles to construct the 95% prediction intervals (95% PIs). We can set the forecasting horizon using the parameter <forecastingperiod1> in the options_forecast.m file. The structure of the options_forecast.m file is described in Supplementary Text 2.

**Fig. 1:**
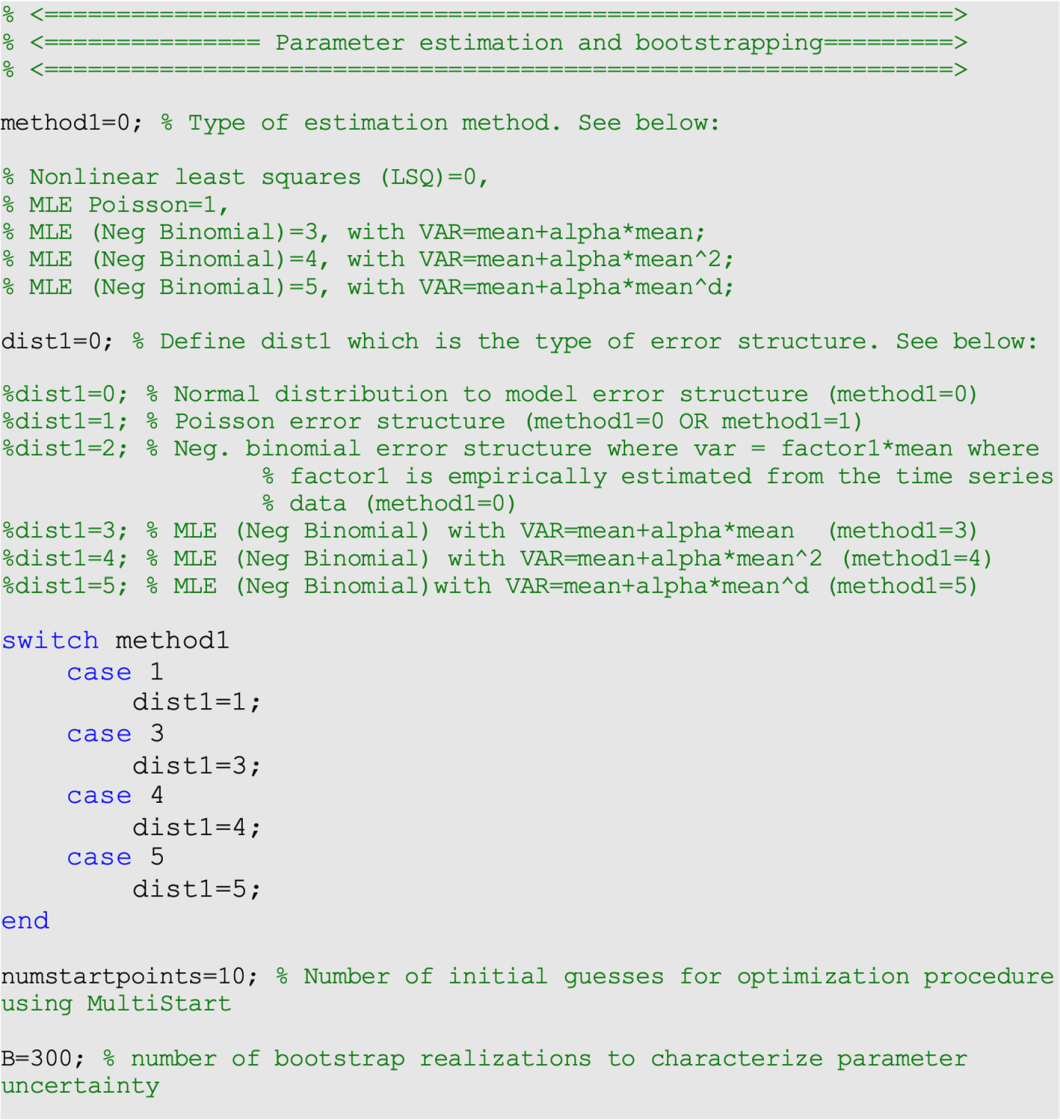
Contents of options.m file, the values of the parameters related to the parameter estimation method and parametric bootstrapping

For the COVID-19 case data employed for illustration purposes, we fit the models by the nonlinear least squares method assuming a normal error structure (i.e., <method1> =0 and <dist1>=0) (Fig. 1).

### Sub-epidemic wave model

We use a spatial wave model with up to 5 parameters that aggregate linked overlapping sub-epidemics [13]. This sub-epidemic framework can characterize diverse epidemic patterns, including the epidemic plateaus, where the epidemic stabilizes at a high level for an extended period and the epidemic waves have multiple peaks. The strength (e.g., weak vs. strong) of their overlap determines when the next sub-epidemic is triggered and is controlled by the onset threshold parameter, ${C}_{thr}$. The mathematical equation for the sub-epidemic building block is the 3-parameter generalized-logistic growth model (GLM), which is specified by setting the parameter <flag1>=1 in the options.m file. This growth model has performed well in short-term forecasts of single outbreak trajectories for different infectious diseases, including COVID-19 [22–24]. Alternative growth equations to model the sub-epidemic building block include the 3-parameter Richards model (<flag1>=4) and the 2-parameter logistic growth model (<flag1>=2). The following differential equation gives the generalized-logistic growth model (GLM):

$$\frac{dC\left(t\right)}{dt}={C}^{{\prime }}\left(t\right)=r{C}^{p}\left(t\right)\left(1-\frac{C\left(t\right)}{{K}_{0}}\right),$$

where $C\left(t\right)$ denotes the cumulative curve at time *t*, and $\frac{dC\left(t\right)}{dt}$ describes the epidemic’s incidence curve over time *t*. The positive parameter $r$ denotes the growth rate per unit of time, ${K}_{0}$ is the final outbreak size, and $p \in \left[\text{0,1}\right]$ is the “scaling of growth” parameter which allows the model to capture early sub-exponential and exponential growth patterns. If $p=0$, this equation describes a constant incidence over time, while $p=1$ indicates that the early growth phase is exponential. Intermediate values of $p\hfill(0 <p<1)$ describe early sub-exponential (e.g., polynomial) growth dynamics. The sub-epidemic wave model consists of a system of coupled differential equations:

$$\frac{d{C}_{i}\left(t\right)}{dt}=r{A}_{i-1}\left(t\right){{C}_{i}\left(t\right)}^{p}\left(1-\frac{{C}_{i}\left(t\right)}{{K}_{i}}\right).$$

Here, ${C}_{i}\left(t\right)$ is the cumulative number of infections for sub-epidemic $i$, and ${K}_{i}$ is the size of the ${i}_{th}$ sub-epidemic where $i=1,\dots ,n$. Starting from an initial sub-epidemic size ${K}_{0}$, the size of consecutive sub-epidemics ${K}_{i}$ decline at the rate *q* following an exponential or power-law function as described below. Hence, a total of 5 parameters $(r,p,{C}_{thr}, {q,K}_{0})$ for $i=1,\dots ,n$ are needed to characterize a sub-epidemic wave composed of two or more sub-epidemics.

The onset timing of the subsequent $${\left(i+1\right)}_{th}$$ sub-epidemic is determined by the indicator variable $${A}_{i}\left(t\right)$$. This results in a coupled system of sub-epidemics where the $${(i+1)}_{th }$$ sub-epidemic is triggered when the cumulative curve for the $${i}_{th}$$ sub-epidemic exceeds a total of $${C}_{thr}$$. The sub-epidemics *overlap* because the ($$i+1{)}_{th}$$sub-epidemic takes off before the $${i}_{th}$$ sub-epidemic completes its course. That is,

$$A_i\left(t\right)=\left\{\begin{array}{lc}1&C_i\left(t\right)>C_{thr}\\0&Otherwise\end{array},\right.i=1,2,\dots,n-1.$$

The threshold parameters are defined so that 1 $\le {C}_{thr}<{K}_{0}$ and ${A}_{0}\left(t\right)=1$ for the first sub-epidemic. The maximum number of sub-epidemics considered in the epidemic wave trajectory is specified using parameter <npatches_fixed> in the options.m file. Here, we set <npatches_fixed>=3. The initial number of cases is given by ${C}_{1}\left(0\right)={I}_{0}$, where ${I}_{0}$ is the initial number of cases in the observed data.

In this framework, the size of the subsequent ${i}_{th}$ sub-epidemic (${K}_{i}$) remains steady or declines due to the effects of behavior changes or interventions. We consider both exponential and inverse decline functions to model the size of consecutive sub-epidemics described below.

### Exponential decline of sub-epidemic sizes

If consecutive sub-epidemics follow exponential decline, then ${K}_{i}$ is given by:

$${K}_{i}={K}_{0}{e}^{-q(i-1)},$$

where *K*_*0*_ is the size of the initial sub-epidemic ${(K}_{1}={K}_{0})$. If $q=0$, the model predicts an epidemic wave composed of sub-epidemics of the same size. When $q>0$, the epidemic wave is composed of a finite number of sub-epidemics given by ${n}_{tot}$ which is a function of ${C}_{thr}, {q, \text{and} K}_{0}$ as follows:

$${n}_{tot}=\left\lfloor-\frac{1}{q}\text{ln}\left(\frac{{C}_{thr}}{{K}_{0}}\right)+1\right\rfloor.$$

where the brackets $\lfloor *\rfloor$ denote the largest integer that is smaller than or equal to $*$. The total size of the epidemic wave composed of ${n}_{tot}$ overlapping sub-epidemics has the following closed-form solution:

$${K}_{tot}=\sum\nolimits _{i=1}^{{n}_{tot}}{K}_{0}{e}^{-q(i-1)}=\frac{{K}_{0}(1-{e}^{-q{n}_{tot}})}{1-{e}^{-q}}.$$

The exponential sub-epidemic decline function can be selected by setting the parameter <typedecline2>=1 in the options.m file.

### Power-law decline of sub-epidemic sizes

If consecutive sub-epidemics decline according to the inverse function, we have:

$${K}_{i}={K}_{0}{\left(\frac{1}{i}\right)}^{q}.$$

When $\text{q}>0$, the total number of sub-epidemics ${n}_{tot}$ comprising the epidemic wave is finite and given by:

$${n}_{tot}=\left\lfloor{\left(\frac{{C}_{thr}}{{K}_{0}}\right)}^{- \frac{1}{q}}\right\rfloor.$$

The total size of an epidemic wave is given by the aggregation of ${n}_{tot}$ overlapping sub-epidemics:

$${K}_{tot}=\sum\nolimits _{i=1}^{{n}_{tot}}{K}_{0}{\left(\frac{1}{i}\right)}^{q}.$$

The power-law sub-epidemic decline function can be selected by setting the parameter <typedecline2> = 2 in the options.m file. Selecting the type of decline function that yields the best fit to the data is also possible by setting the parameter,

<typedecline2>=[1 2].

### Fixed sub-epidemic onset

We can also consider sub-epidemic wave models with a fixed onset time at 0. In this case, all sub-epidemics start at time 0, and the threshold parameter ${C}_{thr}$ drops from the model. We use parameter <onset_fixed> in the options.m file to specify whether the onset timing of the sub-epidemics is fixed at time 0 (<onset_fixed>=1) or not (<onset_fixed>=0).

### Top-ranked sub-epidemic models

To select the top-ranked sub-epidemic models, we analyze the Akaike information criterion ($AI{C}_{c}$) values of the set of best-fit sub-epidemic wave models with different values of ${C}_{thr}$. The ${AIC}_{c}$ is given by [25, 26]:

$${AIC}_{c}=-2log\left(likelihood\right)+2 m+\frac{2 m\left(m+1\right)}{{n}_{d}-m-1},$$

where $m$ is the number of model parameters, and ${n}_{d}$ is the number of data points. Specifically for normal distribution, the ${AIC}_{c}$ is

$${AIC}_{c}={n}_{d}log\left(SSE\right)+2 m+\frac{2 m\left(m+1\right)}{{n}_{d}-m-1},$$

where $SSE=\sum _{j=1}^{{n}_{d}}{(f\left({t}_{j},\widehat{{\Theta }}\right)-{y}_{{t}_{j}})}^{2}$ is the sum of squared errors, $m$ is the number of model parameters including parameter ${C}_{thr}$. Parameter <topmodelsx> in the options.m file is used to specify the number of top-ranked models that will be generated and used to derive ensemble models.

**Fig. 2:**
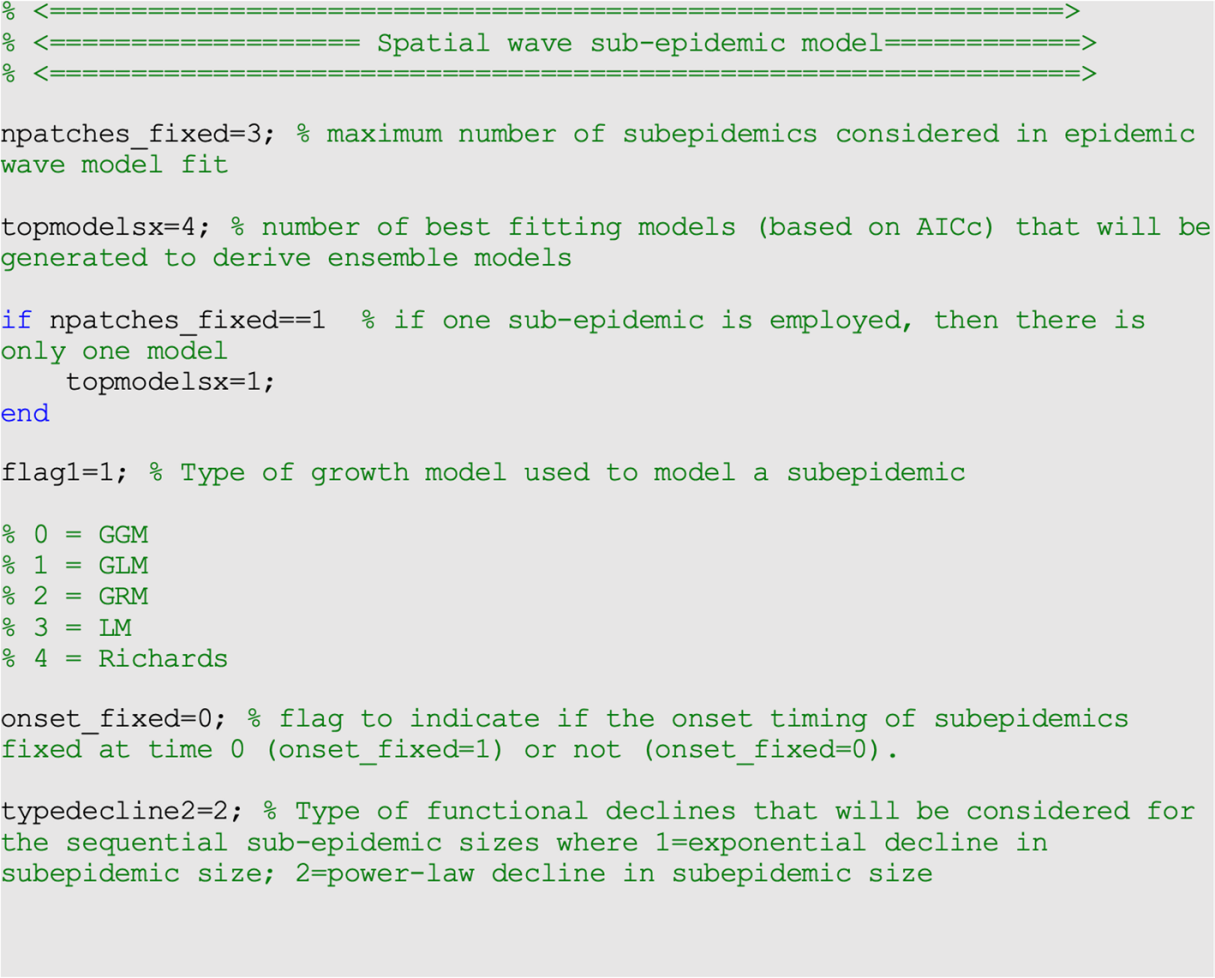
Contents of options.m file, the values of the parameters related to the sub-epidemic wave model and the number of top-ranked sub-epidemic wave models

To illustrate the methodology, we set <onset_fixed>=0, < typedecline2 > = 2 (power-law decline) and analyzed four top-ranking sub-epidemic models (<topmodelsx> = 4). The top-ranking models are used to construct three ensemble sub-epidemic models, which we refer to as: Ensemble(2), Ensemble(3), and Ensemble(4) (Fig. 2).

#### Plotting simulations of the spatial wave sub-epidemic model

Before fitting the growth model to the data, it is useful to check that the selected model yields simulations broadly consistent with the range of the time series data by generating model simulations with different parameter values. For example, if data show systematic differences that contrast with the model solutions, it may suggest that the model is not the best choice for the data at hand.

The function plot_SW_subepidemic.m can be used to plot model solutions where the user provides the type of growth model by passing parameter <flag1> (generalized-logistic growth model, Richards, Gompertz, etc.), the model parameter values, and the initial conditions as passing input parameters to the function in the following order: <flag1>, $r$, $p$, $a,K,q,n, {C}_{thr},$ <typedecline1>, $C\left(0\right)$, and finally the duration of the simulation. For example, the following call plots a simulation of the spatial wave sub-epidemic model using as building block the generalized logistic growth model (< flag1 > = 1) and the following model parameter values: $r=0.18,p=0.18,K=1000,q=0.24,n=8,{C}_{thr}=50$. The initial condition $C\left(0\right)=5$, and the total duration of the simulation is set at 200.

>> plot_SW_subepidemic (1,0.18,0.9,[],1000,0.24,8,50,1,5,200)

**Fig. 3:**
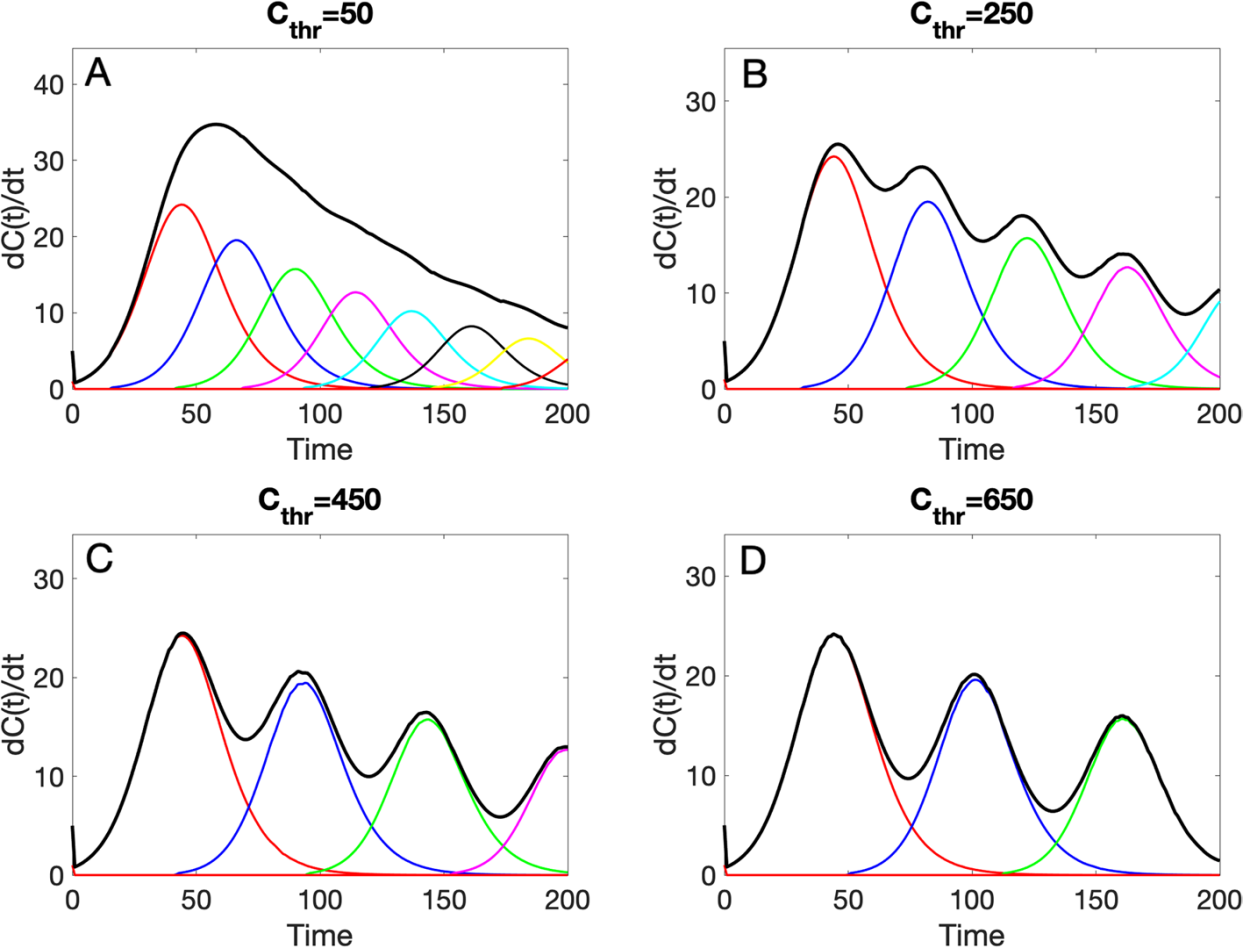
Four representative profiles of the spatial wave sub-epidemic model where the sub-epidemic building block is modeled using the generalized logistic growth model and characterized by the following parameters: $r=0.18,p=0.18,K=1000,q=0.24,n=8$, and the ${C}_{thr}$ value is varied with values: **A** 50, **B** 250, **C** 450, **D** 650. An exponential function is used to model the decline of sub-epidemic sizes (< typedecline1 >=1). The solid black line corresponds to the overall aggregated curve whereas the individual sub-epidemics are shown in different colors

Of note, in the above call, the value of parameter $a$ is passed empty ([]) since the generalized logistic growth model does not use this parameter. This function will generate a figure (Fig. 3A) that shows the corresponding model solution $dC\left(t\right)/dt$. Additional representative simulations with other values of the ${C}_{thr}$ are shown in Fig. 3.

In the next section, we describe four comprehensive performance metrics that can be used to assess both calibration and forecasting performance. Specifically, the mean absolute error (MAE) and the mean squared error (MSE) are used to assess the performance of point forecasts, while the coverage of the 95% prediction interval (95% PI) and the weighted interval score (WIS) evaluate the performance of distributional forecasts by accounting for uncertainty in model fit and predictions.

## Performance metrics

To assess the performance of the models during the calibration or forecasting periods, we used four performance metrics: the mean absolute error (MAE), the mean squared error (MSE), the coverage of the 95% prediction intervals (95% PI), and the weighted interval score (WIS) [27]. While it is possible to generate *h*-time units ahead forecasts of an evolving process, those forecasts looking into the future can only be evaluated until sufficient data for the *h*-time units ahead has been collected. In the options_forecast.m file, the parameter <getperformance> is a Boolean variable (0/1) to indicate whether the user wishes to compute the performance metrics of the forecasts when sufficient data is available.

The *mean absolute error* (MAE) is given by:

$$\text{MAE}\text{ }\text{= }\frac{1}{N}\sum\nolimits _{h=1}^{N}\left|f\left({t}_{h},\widehat{\varTheta }\right)-{y}_{{t}_{h}}\right|,$$

where ${t}_{h}$are the time points of the time series data [28], and *N* is the calibration or forecasting period length. Similarly, the *mean squared error* (MSE) is given by:

$$\text{MSE}\text{ }\text{= }\frac{1}{N}{\sum }_{h=1}^{N}(f\left({t}_{h},\widehat{\varTheta }\right)-{y}_{{t}_{h}}{)}^{2} ,$$

where ${t}_{h}$are the time points of the time series data [28], and N is the calibration or forecasting period length. The coverage of the *95% prediction interval* (PI) corresponds to the fraction of data points that fall within the 95% PI, calculated as

$$95\%{\;\mathrm{PI}\;\mathrm{coverage}\;=\;\frac1N}\sum\nolimits_{h=1}^N1\{{Y_t}_h>L_{t_h}\cap Y_{t_h}<U_{t_h}\}$$

where ${L}_{{t}_{h}}$and ${U}_{{t}_{h}}$ are the lower and upper bounds of the 95% PIs, respectively, ${Y}_{{t}_{h}}$are the data and 1 is an indicator variable that equals 1 if ${Y}_{{t}_{h}}$is in the specified interval and 0 otherwise.

The *weighted interval score* (WIS) [27, 29], which is a proper score recently embraced for quantifying model forecasting performance in epidemic forecasting studies [30–33], provides quantiles of predictive forecast distribution by combining a set of Interval Scores (IS) for probabilistic forecasts. An IS is a simple proper score that requires only a central $(1-\alpha )\times 100\%$ PI [27] and is described as

$${IS}_{\alpha }\left(F,y\right)=\left(u-l\right)+\frac{2}{\alpha }\times \left(l-y\right)\times 1\left(y<l\right)+\frac{2}{\alpha }\times \left(y-u\right)\times 1\left(y>u\right) .$$

In this Eq. 1 refers to the indicator function, meaning that $1\left(y<l\right)=1$ if $y<l$ and 0 otherwise. The terms $l$ and $u$ represent the $\frac{\alpha }{2}$ and $1-\frac{\alpha }{2}$ quantiles of the forecast *F*. The IS consists of three distinct quantities:

1. The sharpness of $F,$ given by the width $u-l$ of the central $\left(1-\alpha \right) \times 100\%$ PI.
2. A penalty term $\frac{2}{\alpha }\times \left(l-y\right)\times 1\left(y<l\right)$ for the observations that fall below the lower end point $l$ of the $\left(1-\alpha \right)\times 100\%$ PI. This penalty term is directly proportional to the distance between $y$ and the lower end $l$ of the PI. The strength of the penalty depends on the level $\alpha .$
3. An analogous penalty term $\frac{2}{\alpha }\times \left(y-u\right)\times 1\left(y>u\right)$ for the observations falling above the upper limit $u$ of the PI.

To provide more detailed and accurate information on the entire predictive distribution, we report several central PIs at different levels $\left(1-{\alpha }_{1}\right)<\left(1-{\alpha }_{2}\right)<\dots <\left(1-{\alpha }_{K}\right)$ along with the predictive median, $\stackrel{\sim}{y}$, which can be seen as a central prediction interval at level $1-\alpha_0\rightarrow0$. This is referred to as the WIS, and it can be evaluated as follows:

$$WI{S}_{{\alpha }_{0:K}}\left(F,y\right)=\frac{1}{K+\frac{1}{2}}.\left({w}_{0}.\left|y-\stackrel{\sim}{y}\right|+\sum\limits _{k=1}^{K}{w}_{k}.I{S}_{{\alpha }_{k}}\left(F,y\right)\right),$$

where, ${w}_{k}=\frac{{\alpha }_{k} }{2}$for $k=\text{1,2},\dots .K$ and ${w}_{0}=\frac{1}{2}$. Hence, WIS can be interpreted as a measure of how close the entire distribution is to the observation in units on the scale of the observed data [31, 34].

### Doubling times

Doubling times characterize the sequence of times at which the cumulative incidence doubles. We denote the times at which cumulative incidence doubles by ${t}_{{d}_{j}}$, such that ${2 C(t}_{{d}_{j}})={C(t}_{{d}_{j+1}})$ where ${t}_{{d}_{0}}=0, C\left( {t}_{{d}_{0}}\right)={C}_{0}$, $j=\text{1,2},3,\dots ,{n}_{g}$ and ${n}_{g}$ is the total number of times cumulative incidence doubles [35]. The actual sequence of “doubling times” is defined as follows:

$${d}_{j}={\varDelta t}_{{d}_{j}}={t}_{{d}_{j}}-{t}_{{d}_{j-1}} \text{w}\text{h}\text{e}\text{r}\text{e}\ j=\text{1,2},3,\dots ,{n}_{g}.$$

For exponential growth, doubling times remain invariant and are given by $\left(ln2\right)/r$, whereas the doubling times increase when the growth pattern follows sub-exponential growth [36]. We can characterize the doubling times and their uncertainty from the best-fit model $f\left(t,\widehat{{\Theta }}\right)$ [37]. We can evaluate the uncertainty of the sequence of doubling times and the overall doubling time using the model parameter estimates derived from bootstrapping $\left({\widehat{{\Theta }}}_{b}\right)$, where $b=\text{1,2},3,\dots ,B$. That is, ${d}_{j}\left({\widehat{{\Theta }}}_{b}\right)$ provides a sequence of doubling times for a set of bootstrap parameter estimates, ${\widehat{{\Theta }}}_{b}$, where $b=\text{1,2},3,\dots ,B$. We can use these curves to derive 95% CIs for the sequence of doubling times and quantify the probability of observing a given number of doublings.

### Constructing ensemble forecasts from top-ranking models

**Fig. 4:**
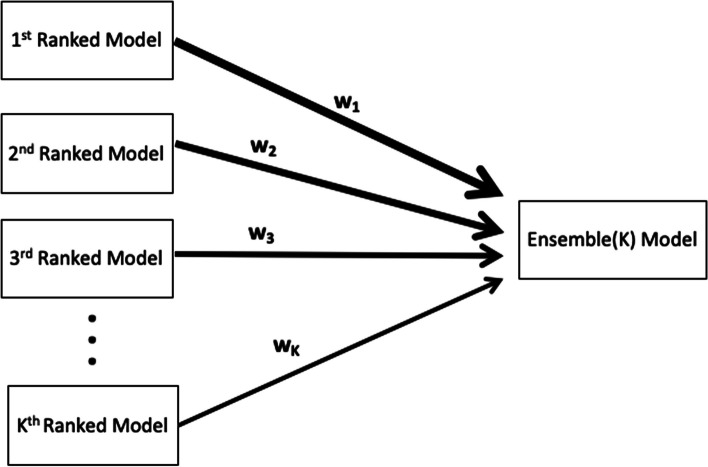
Schematic diagram of the construction of the ensemble model from the weighted combination of the highest-ranking sub-epidemic models as deemed by the ${AIC}_{{c}_{k}}$ for the $k$-th model where ${ AIC}_{{c}_{1}}\le \dots \le {AIC}_{{c}_{K}}$ and $k$ = 1, …, *K*. An ensemble derived from the top-ranking $K$ models is denoted by Ensemble(K)

Ensemble models that combine the strength of multiple models may exhibit significantly enhanced predictive performance (e.g [11, 17, 38, 39]). An ensemble model derived from the top-ranking $I$ models is denoted by the Ensemble(1), illustrated in Fig. 4. Thus, Ensemble(2) and Ensemble(3) refer to the ensemble models generated from the combination of the top-ranking 2 and 3 models, respectively. The ensemble models can be derived from the unweighted (equal weights across contributing individual models) or a weighted combination of the highest-ranking sub-epidemic models based on the quality of fit as deemed by the ${AIC}_{{c}_{i}}$for the *i*-th model where ${AIC}_{{c}_{1}}\le \dots \le {AIC}_{{c}_{I}}$ and $i$ = 1, …, *I*. In this case, we compute the weight ${w}_{i}$for the *i*-th model, $i$ = 1, …, *I*, where $\sum {w}_{i}$ = 1 as follows:

$${w}_{i}=\frac{\frac{1}{{AIC}_{{c}_{i}}}}{\frac{1}{{AIC}_{{c}_{1}}}+\frac{1}{{AIC}_{{c}_{2}}}+\dots +\frac{1}{{AIC}_{{c}_{I}}}} \text{f}\text{o}\text{r}\ \text{a}\text{l}\text{l}\ i=\text{1,2},\dots ,I,$$

and hence $${\text{w}}_{\text{I}}\le \dots {\le \text{w}}_{1}$$.

The estimated mean curve of daily COVID-19 cases for the Ensemble(*I*) model is:

$${f}_{ens\left(I\right)}\left(t\right)=\sum_{i=1}^{I}{w}_{i}{f}_{i}\left(t,{\widehat{{\Theta }}}^{(i)}\right),$$

where given the training data, $\widehat\Theta^{\left(i\right)}$ denotes the set of estimated parameters, and $f_i\left(t,\;\widehat\Theta\right)^{\left(i\right)}$ denotes the estimated mean curve of daily COVID-19 cases, for the *i*-th model. Accordingly, we compute the weighted average and sample the bootstrap realizations of the forecasts for each model to construct the 95% CI or PI using the 2.5% and 97.5% quantiles [17]. Alternatively, we can set the ensemble weights based on different calibration performance metrics for the top-ranked models. For instance, we can make the ensemble weights proportional to the relative likelihood ($l$) rather than the reciprocal of the $AI{C}_{c}$. Let ${AIC}_{min}$ denote the minimum $AIC$ from the set of models. The relative likelihood of model *i* is given by ${l}_{i}={e}^{({(AIC}_{min}-{AIC}_{i})/2)}$ [40]. We compute the weight ${w}_{i}$ for the $i$-th model where $\sum {w}_{i}$ = 1 as follows:

$${w}_{i}=\frac{{l}_{i}}{{l}_{1}+{l}_{2}+\dots +{l}_{I}} \text{f}\text{o}\text{r} \text{a}\text{l}\text{l} i=\text{1,2},\dots ,I,$$

and hence $${w}_{I}\le \dots {\le w}_{1}$$.

In the options_forecast.m file, we can specify four types of ensemble weights using <weight_type1>. Specifically, unweighted (<weigth_type1>=-1), weighted according to the $AI{C}_{c}$ (<weight_type1>=0), weighted based on the relative likelihood (weight_type1=1), weighted based on the reciprocal of the WIS metric of the calibration period (<weight_type1>=2).

**Fig. 5:**
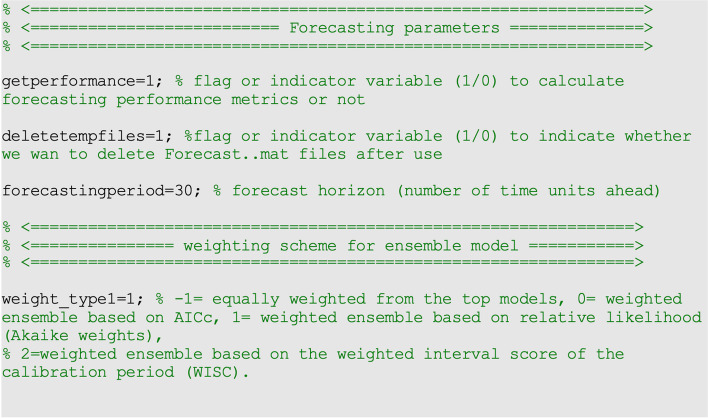
Contents of options_forecast.m file, that specify the parameters related to the epidemic forecasts including the forecasting horizon and the type of ensemble weights (e.g., unweighted, weighted based on $AI{C}_{c}$, weighted based on the relative likelihood of the models, and weighted based on the WIS of the calibration period)

In the options_forecast.m file, we can specify the parameters related to the epidemic forecasts, including the forecasting horizon and the type of ensemble weights (Fig. 5).

## Results and discussion

### The dataset

The time series data file is a text file with the extension *.txt in the input folder. The data file can contain one or more incidence time series (one per column). Each column corresponds to the incidence curve over time for each epidemic corresponding to a different area/group. For instance, each column could contain time series data corresponding to different U.S. states or countries worldwide. In the options.m file, a specific data column can be accessed for inference using the parameter <outbreakx>. If the time series file contains cumulative incidence count data, the name of the file containing the time series data starts with “cumulative” according to the following format:

**cumulative** -< cadtemporal>-<caddisease>-<datatype>-<cadregion>-<caddate1> .**txt**.

where <cadtemporal> is a string parameter that indicates the temporal resolution of the data (e.g., daily, weekly, yearly). Parameter <caddisease> is a string used to indicate the name of the disease related to the time series data, <datatype> is a string parameter indicating the nature of the data (e.g., cases, deaths, and hospitalizations), whereas <cadregion> is a string parameter indicating the geographic region of the time series contained in the file (New York, USA, World, Asia, Africa). Finally, <caddate1> is a string to indicate the date for the most recent observation in the data file with the format: mm-dd-yyyy.

**Fig. 6:**
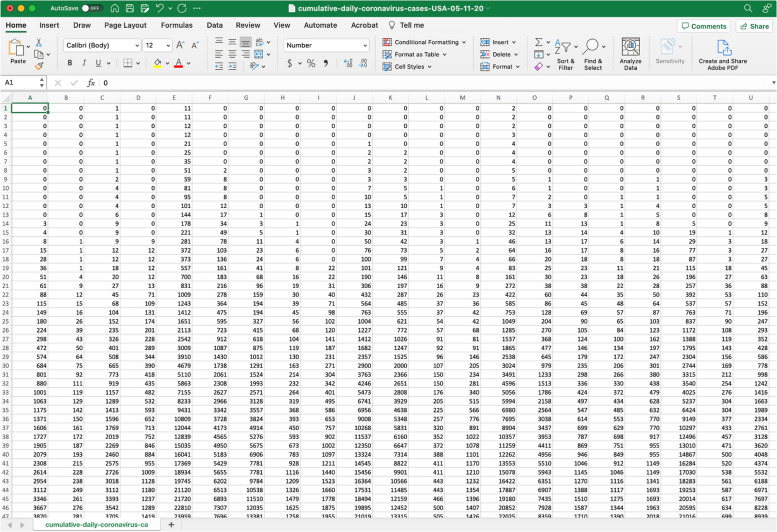
Example data file named cumulative-daily-coronavirus-cases-USA-05-11-2020.txt located in the input folder. A partial view in Excel of the contents of the data file is shown

To illustrate the methodology presented in this tutorial paper, we used daily COVID-19 cases reported in the USA from the publicly available data tracking system of the Johns Hopkins Center for Systems Science and Engineering (CSSE) [41]. The data is also publicly available in the GitHub repository [42]. An example of a data file that we will use in this tutorial is provided in Fig. 6.

If the time series file contains incidence data, the name of the data file does not start with the word ‘cumulative’ and follows the format:

*<cadtemporal>-<caddisease>-<datatype>-<cadregion>-<caddate1>*.**txt**

For example: daily-coronavirus-cases-USA-05-11-2020.txt

**Fig. 7:**
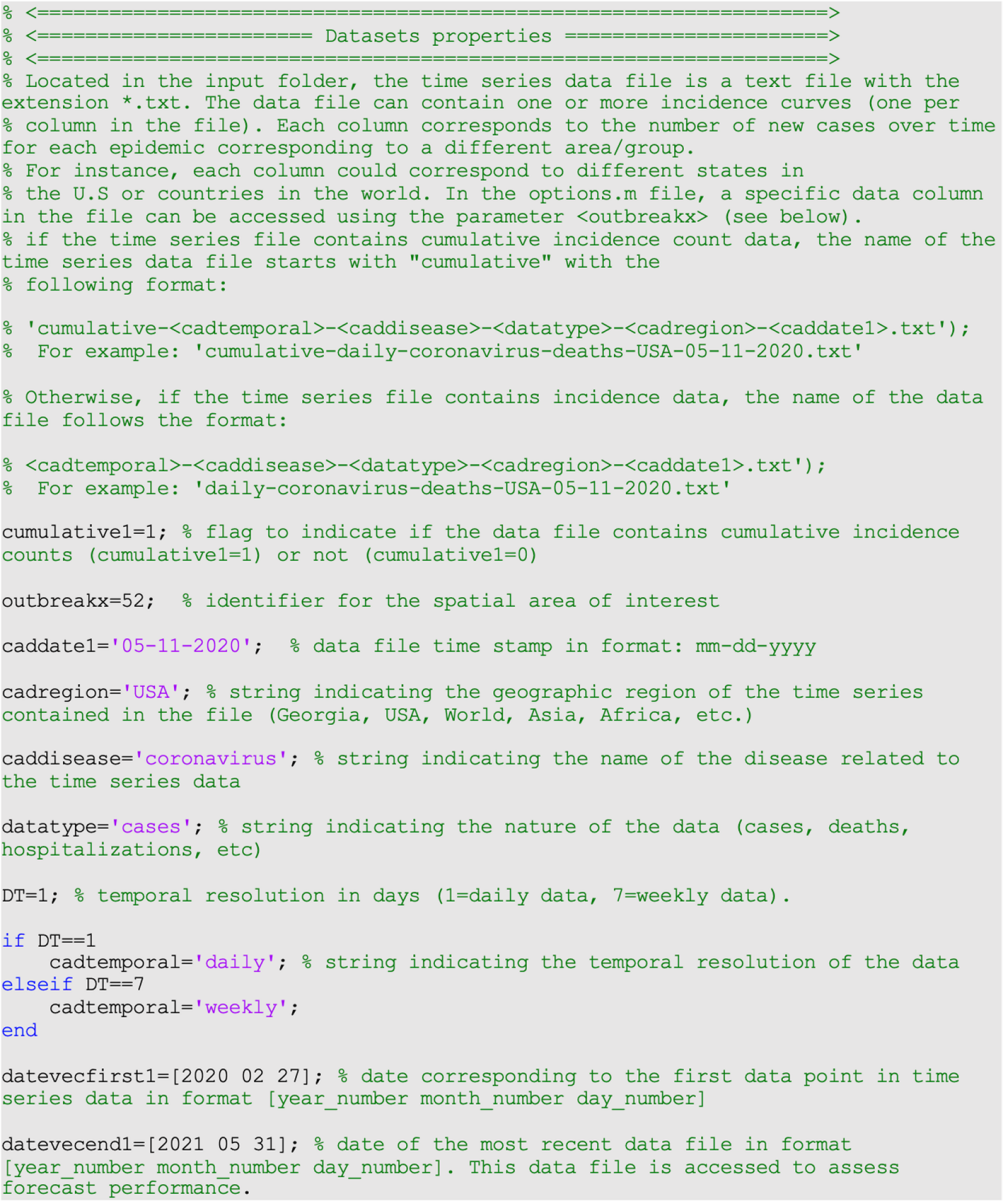
Contents of options.m file, and the values of the parameters related to the data including the temporal resolution of the time series data

In the options.m file, the parameter <datevecfirst1> is a 3-value vector that specifies the date corresponding to the first data point in time series data in the format: [yyyy mm dd]. Similarly, the parameter <datevecend1> is a 3-value vector that specifies the date of the most recent data file in the format: [yyyy mm dd]. The file.

cumulative-<cadtemporal>-<caddisease>-<datatype>-<cadregion>-**<datevecend1>**.txt

in the input folder with the date **<datevecend1>** contains the most recent time series data and is needed to assess forecast performance. Finally, the parameter <DT> is an integer indicating the temporal resolution of the time series data (e.g., <DT> = 1 for daily data; <DT> = 7 for weekly data) (Fig. 7).

### Data adjustments

#### Data smoothing

To reduce the noise in the original data due to artificial reasons such as the weekend effects, we can smooth out the time series data using the moving average of the time series whereby <smoothfactor1> is a parameter in the options.m file that specifies the span of the moving average (e.g., <smoothfactor1> = 1 implies no smoothing applied to the data). Let

$${y}_{{t}_{j}=}{y}_{{t}_{1}, }{y}_{{t}_{2}},\dots ,{y}_{{t}_{{n}_{d}}}\text{w}\text{h}\text{e}\text{r}\text{e}\ j=\text{1,2},\dots ,{n}_{d}$$

denote the smoothed time series of the epidemic trajectory based on the moving average. Here, ${t}_{j}$are the time points for the time series data, ${n}_{d}$ is the number of observations, and each ${y}_{{t}_{j}},$

$j=1,2,\dots ,{n}_{d}$, correspond to the smoothed time series. We recommend that the user set the average to multiples of seven to reduce the weekend effects in the reported data.

For the daily COVID-19 case data employed for illustration purposes, we set <smoothfactor1> = 7 and smooth out the daily series using a 7-day moving average to reduce the noise in the original data due to artificial reasons such as the weekend effects.

#### Calibration period

To fit the models to the most recent observations in a time series file, we can specify the length of the calibration period whereby <calibrationperiod1> indicates the number of recent data points that will be used to calibrate the models. If <calibrationperiod1> exceeds the length of the time series in the data file, the calibration period is set to the maximum length of the available data.

**Fig. 8:**
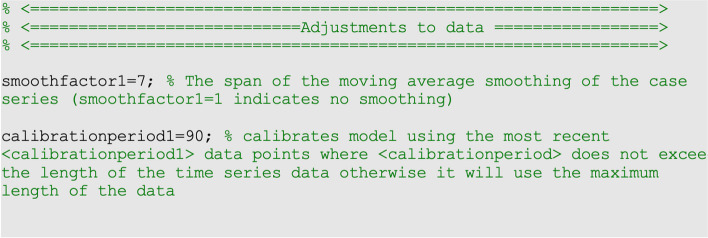
Contents of options.m file, the values of the parameters related to smoothing and calibration period

For illustration purposes, we used a 90-day calibration period (i.e., <calibrationperiod1> = 90) (Fig. 8).

### Fitting the sub-epidemic wave models to data with quantified uncertainty

To fit the sub-epidemic wave models to the data with quantified uncertainty, we need to run the function Run_SW_subepidemicFramework.m. This function uses the input parameters provided by the user in the options.m file. However, the function can also receive <outbreakx> and <caddate1> as passing input parameters while the remaining inputs are obtained from the options.m file.

For example, to fit the ensemble sub-epidemic models to the daily curve of COVID-19 cases in the USA as of the week of ‘05-11-2020’ (data file path: input/cumulative-daily-coronavirus-cases-USA-05-11-2020.txt), we can run the function from MATLAB’s command line window as follows:

>> Run_SW_subepidemicFramework (52,‘05-11-2020’)

This function will generate several output MATLAB files in the output folder. For instance, the following output file contains the fits of the top-ranking models:

ABC-original-npatchesfixed-4-onsetfixed-0-typedecline-2-smoothing-1-daily-coronavirus-cases-USA-state-52-05-11-2020-flag1-1-method-0-dist-0-calibrationperiod-90.mat

Please note that the names of the output files contain the values of the parameters for reference.

The following output files contain the uncertainty characteristics associated with each of the top-ranking models:

- modifiedLogisticPatch-original-npatchesfixed-4-onsetfixed-0-typedecline-2-smoothing-1-daily-coronavirus-cases-USA-state-52-05-11-2020-flag1-1-method-0-dist-0-calibrationperiod-90-**rank-1**.mat
- modifiedLogisticPatch-original-npatchesfixed-4-onsetfixed-0-typedecline-2-smoothing-1-daily-coronavirus-cases-USA-state-52-05-11-2020-flag1-1-method-0-dist-0-calibrationperiod-90-**rank-2**.mat
- modifiedLogisticPatch-original-npatchesfixed-4-onsetfixed-0-typedecline-2-smoothing-1-daily-coronavirus-cases-USA-state-52-05-11-2020-flag1-1-method-0-dist-0-calibrationperiod-90-**rank-3**.mat
- modifiedLogisticPatch-original-npatchesfixed-4-onsetfixed-0-typedecline-2-smoothing-1-daily-coronavirus-cases-USA-state-52-05-11-2020-flag1-1-method-0-dist-0-calibrationperiod-90-**rank-4**.mat

These output internal files are needed to plot model fits, derive parameter estimates, generate short-term forecasts, and quantify the calibration and forecasting performance metrics.

### Plot the mean model fits and quality of fit metrics for the top-ranked models

After running the function Run_SW_subepidemicFramework.m with the desired input parameters, we can use the function plotRankings_SW_subepidemicFramework.m to plot the mean model fits of the top-ranking models including their sub-epidemic profiles and the associated quality of model fit metrics including the $AI{C}_{c}$, the relative likelihood, and the evidence ratio based on the inputs. However, this function can also receive <outbreakx> and <caddate1> as passing input parameters while the remaining inputs are obtained from the options.m file. Running this function from MATLAB’s command line, we have:

>>**plotRankings_SW_subepidemicFramework**(52,‘05-11-2020’)

**Fig. 9:**
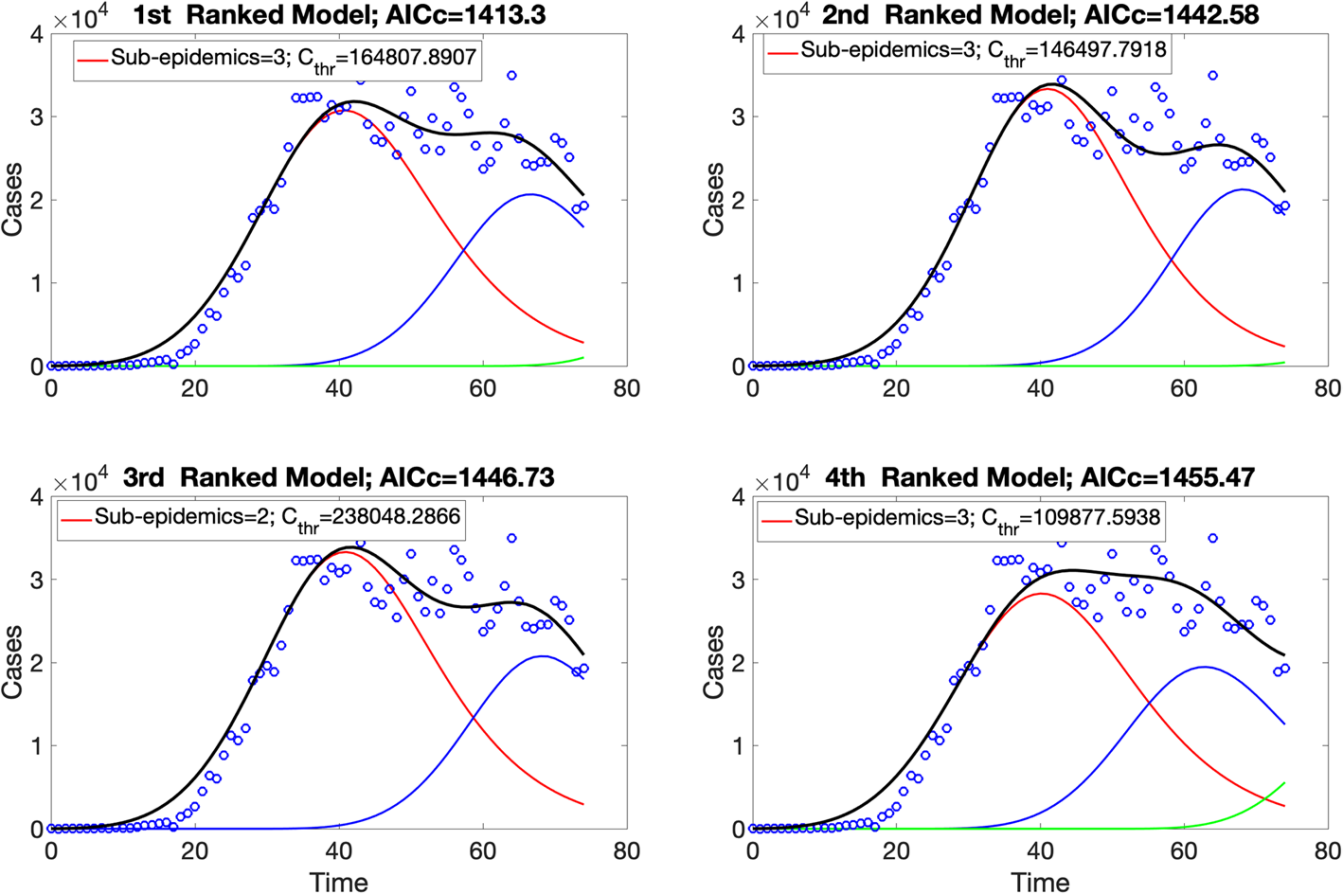
Mean model fits of the top-ranked sub-epidemic models (< topmodelsx>=4 in options.m file) calibrated to the daily curve of COVID-19 cases in the USA from 27-Feb-2020 to 11-May-2020. The solid lines of blue, red, and green correspond to the individual sub-epidemic curves. The solid black line represents the overall aggregated epidemic curve. The legend in each panel indicates the number of sub-epidemics involved in each model and the value of the ${C}_{thr}$parameter

**Fig. 10:**
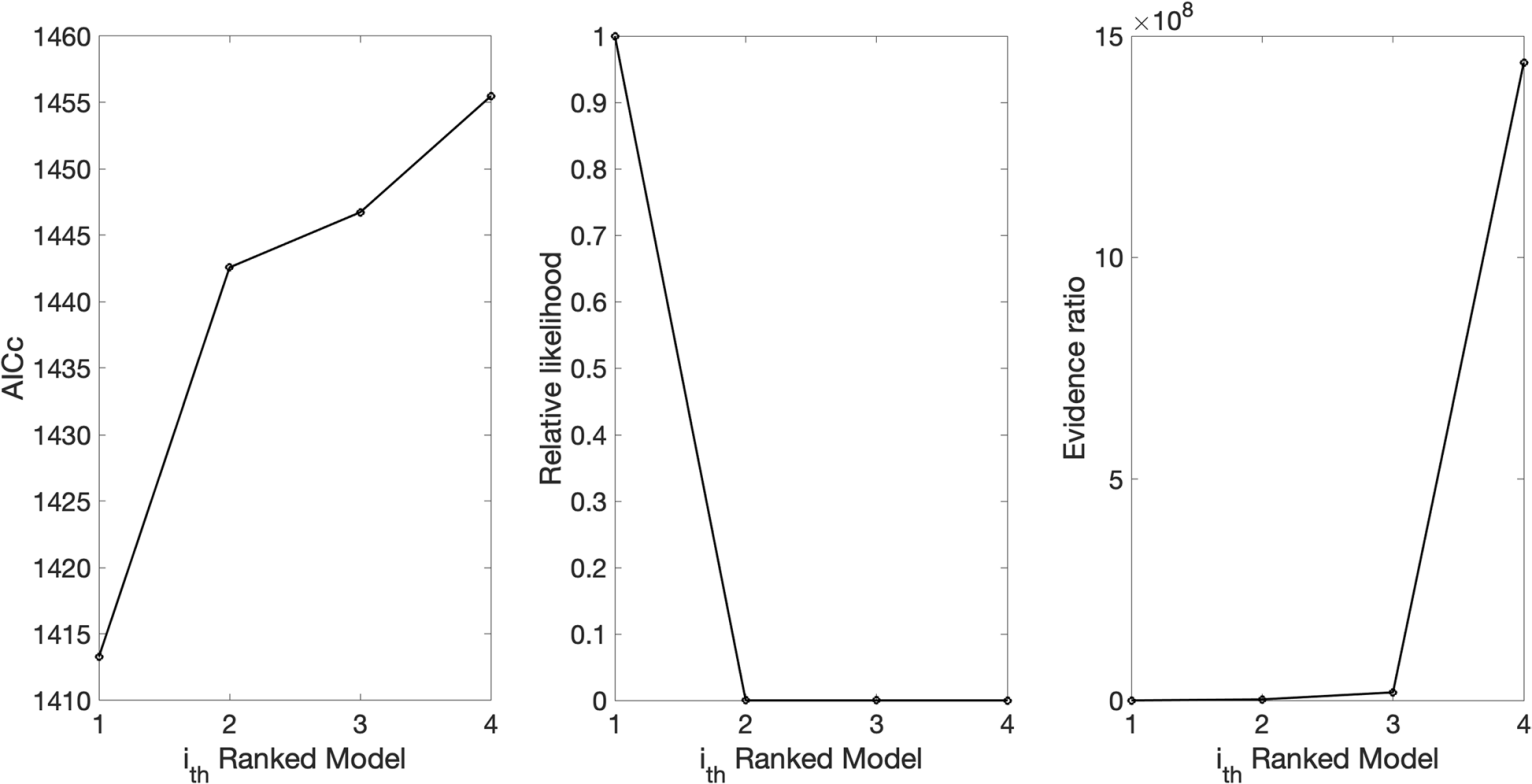
Quality of model fit metrics for the top-ranked sub-epidemic models (<topmodelsx>=4 in options.m file) calibrated to the daily curve of COVID-19 cases in the USA from 27-Feb-2020 to 11-May-2020

Figures 9 and 10 illustrate the outputs obtained from this function call. Figure 9 shows the mean model fits of the top-ranked sub-epidemic models, which indicates that the 1st-ranked model consists of 3 sub-epidemics. In contrast, the 2nd, 3rd, and 4th -ranked sub-epidemic models consist of 2 sub-epidemics. It is important to note that there was severe underreporting of cases during the early phase of the epidemic. The corresponding goodness of fit statistics of the top-ranked models, including the $AI{C}_{c}$, the relative likelihood, and the evidence ratio, are shown in Fig. 10. It also saves the $AI{C}_{c}$ values of the top-ranked models in the following .csv file:

AICc-topRanked-onsetfixed-0-typedecline-2-flag1-1-method-0-dist-0-daily-coronavirus-cases-USA-area-52-05-11-2020.csv.

For comparison, a simpler growth model consisting of a single sub-epidemic (<npatches_fixed> = 1) performs substantially worse ($AI{C}_{c}=1530.4$; Supplementary Fig. 1).

## Plot the model fits, parameter estimates, and performance metrics of the top-ranking models

Using the function plotFit_SW_subepidemicFramework.m, we can plot the fits of the top-ranking models, including their sub-epidemic profiles, parameter estimates, and residual plots based on the inputs indicated in the options.m file. However, this function can also receive <outbreakx> and <caddate1> as passing input parameters while the remaining inputs are obtained from the options.m file.

In addition, this function also plots the empirical distributions of the parameters associated with each of the top-ranking models and the calibration performance metrics (MSE, MAE, 95% P.I., and WIS). Finally, this function also outputs .csv files in the output folder with the calibration performance metrics, the parameter estimates associated with the top-ranking models, the corresponding Monte Carlo standard errors of the parameters, and the estimated sequence of doubling times for each of the top-ranked models. Using the default parameter values indicated in the options.m file, the actual call to this function from MATLAB’s command line follows:

>> **plotFit_SW_subepidemicFramework**

**Fig. 11:**
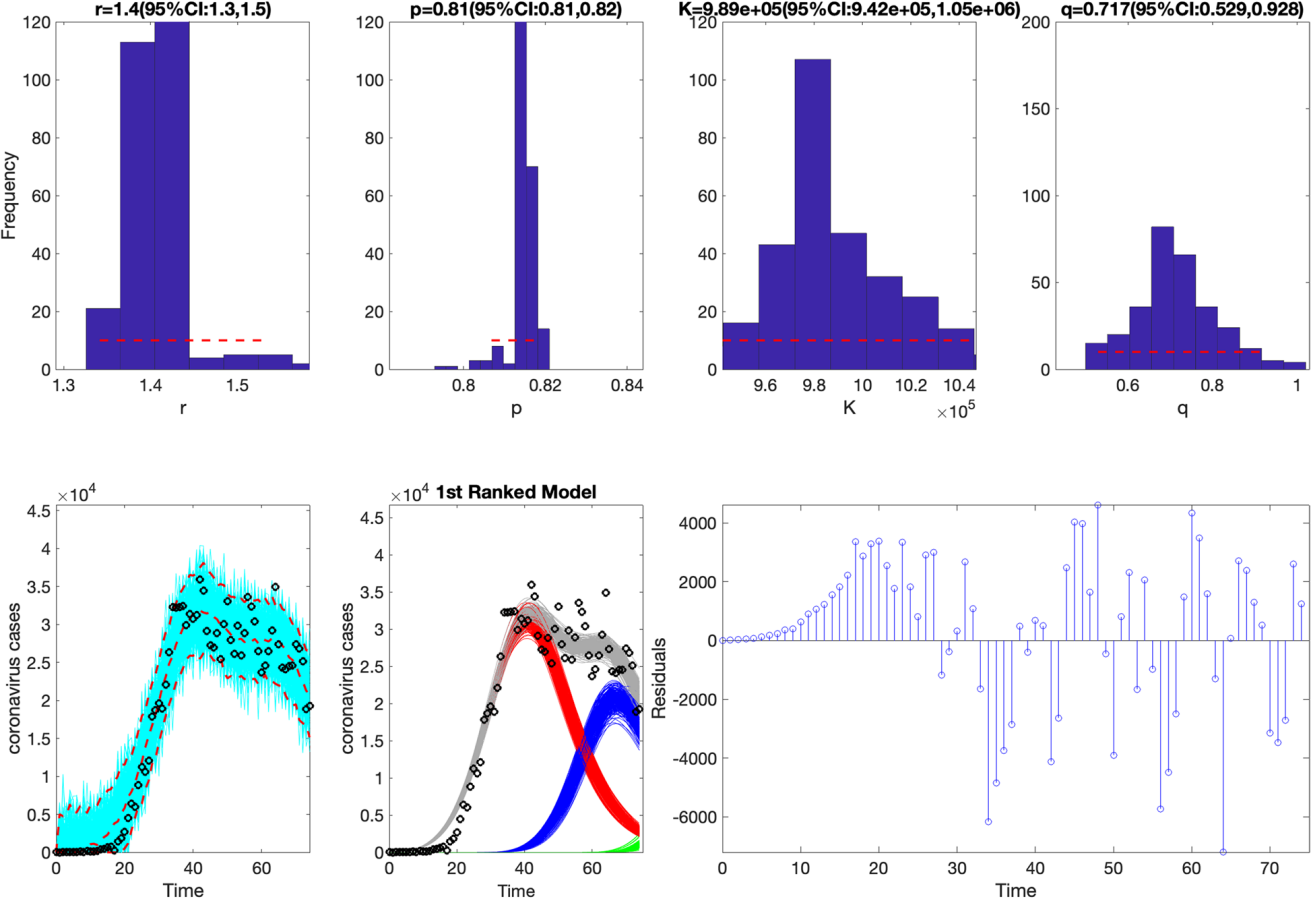
Fit of the 1st-ranked sub-epidemic wave model to the daily curve of COVID-19 cases in the USA from 27-Feb-2020 to 11-May-2020. The model captures the entire epidemic period well, including the broad peak dynamics, by integrating three asynchronous sub-epidemics. The best model fit (solid red line) and 95% prediction interval (dashed red lines) are shown. The cyan curves correspond to the associated uncertainty from individual bootstrapped curves, which are used to derive the 95% prediction intervals. The sub-epidemic mean profiles obtained from the parametric bootstrapping with 300 bootstrap realizations are shown in the center panels. The red, blue, and green curves represent the three sub-epidemic profiles, and the grey curves are the estimated aggregate epidemic trajectories. Black circles correspond to the data points. The empirical distributions of the parameters and the corresponding estimates are shown in the top panels. The residuals are also shown

**Fig. 12:**
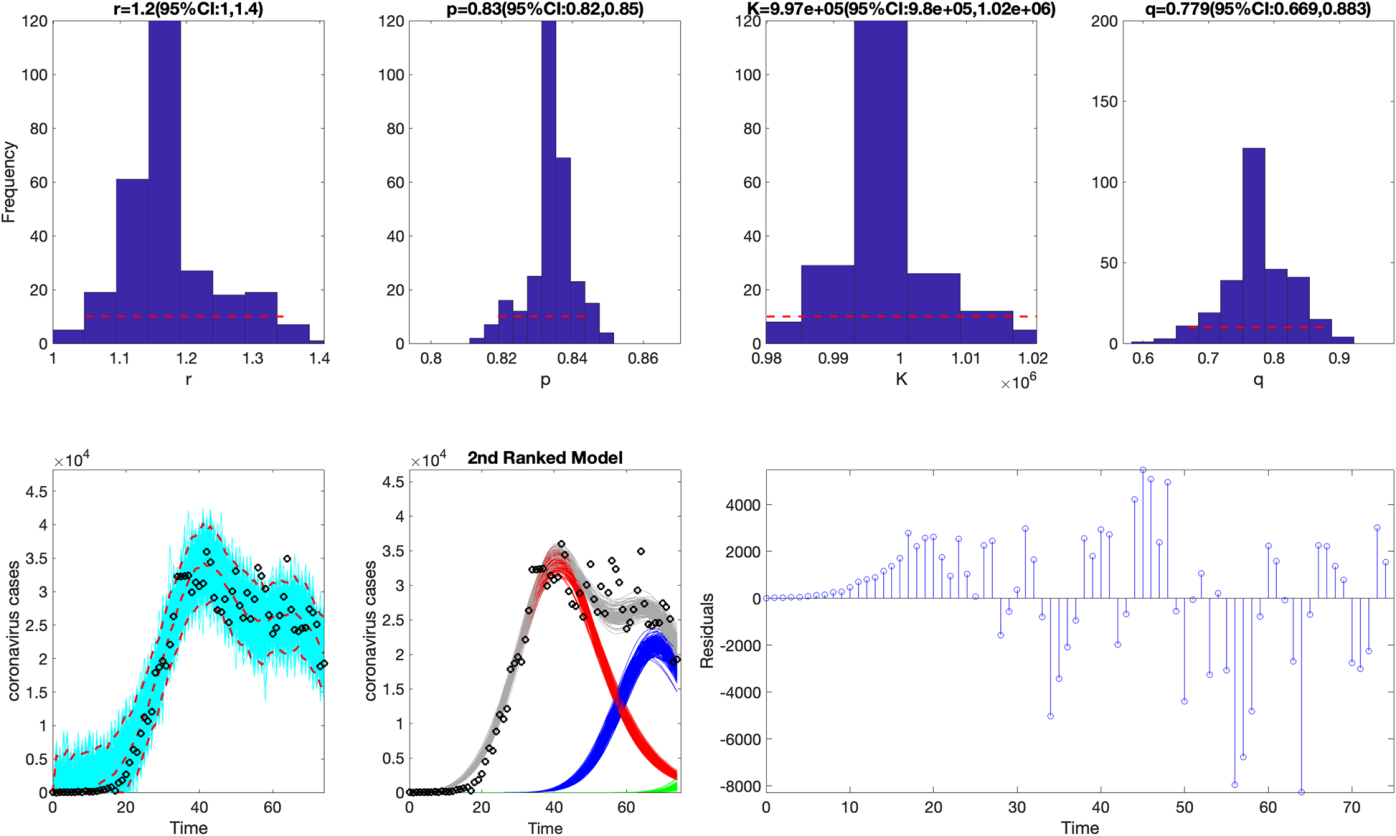
Fit of the 2nd-ranked sub-epidemic wave model to the daily curve of COVID-19 cases in the USA from 27-Feb-2020 to 11-May-2020. The model captures the entire epidemic period well, including the broad peak dynamics, by integrating three asynchronous sub-epidemics. The best model fit (solid red line) and 95% prediction interval (dashed red lines) are shown. The cyan curves correspond to the associated uncertainty from individual bootstrapped curves, which are used to derive the 95% prediction intervals. The sub-epidemic mean profiles obtained from the parametric bootstrapping with 300 bootstrap realizations are shown in the center panels. The red, blue, and green curves represent the two sub-epidemic profiles, and the grey curves are the estimated aggregate epidemic trajectories. Black circles correspond to the data points. The empirical distributions of the parameters and the corresponding estimates are shown in the top panels. The residuals are also shown

**Fig. 13:**
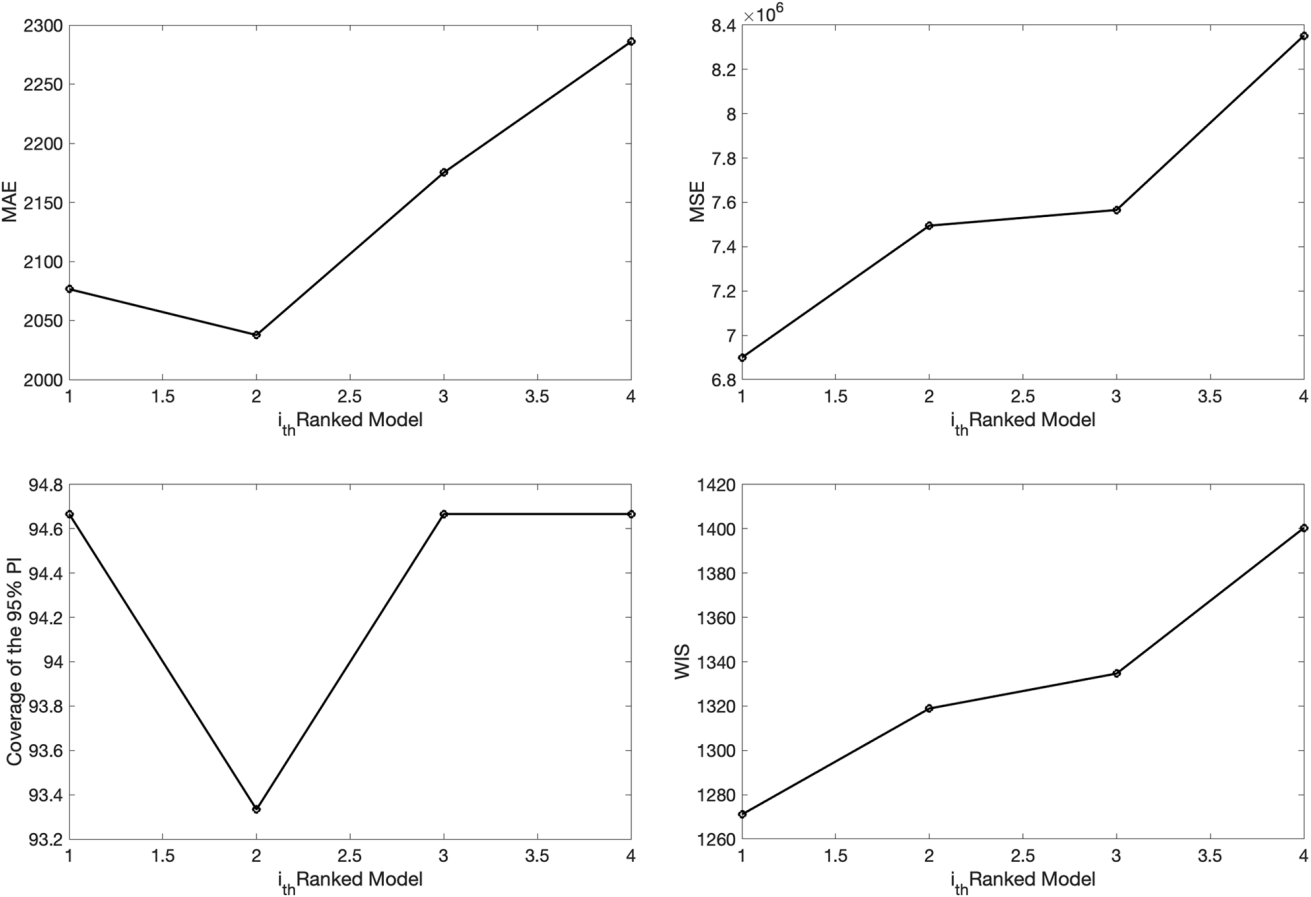
Calibration performance metrics for the top-ranking sub-epidemic wave models fit to the daily curve of COVID-19 cases in the USA from 27-Feb-2020 to 11-May-2020. These metrics are also saved in a .csv data file (‘performance-calibration-topRanked-onsetfixed-0-typedecline-3-flag1-1-method-0-dist-0-horizon-30-daily-coronavirus-cases-USA-area-52-05-11-2020.csv’). For instance, these WIS metrics during the calibration period ranged from ~ 119.7 to ~ 124.8 across the four top-ranked models

Figures 11 and 12 illustrate the outputs from the above call to the function. The fits of the 1st and 2nd ranked sub-epidemic models, including the sub-epidemic profiles and residuals, to the daily curve of COVID-19 cases are shown in Figs. 11 and 12. These models yield a similarly good fit to the data. The figures also include the empirical distribution of the parameter estimates. These parameter estimates are well identified as the confidence intervals lie in a well-defined range of values [13]. The calibration performance metrics capturing the quality of fit of the top-ranked sub-epidemic models are also displayed in Fig. 13. For instance, this figure indicates that the coverage of the 95% PIs varied little between ~ 93% and 95% for the top-ranked models. This function will store the following .csv files in the output folder:

1. Model parameter estimates:

parameters-topRanked-onsetfixed-0-typedecline-2-flag1-1-method-0-dist-0-daily-coronavirus-cases-USA-area-52-05-11-2020.csv

2) Monte Carlo standard errors:

MCSES-topRanked-onsetfixed-0-typedecline-2-flag1-1-method-0-dist-0-daily-coronavirus-cases-USA-area-52-05-11-2020.csv

3) Calibration performance metrics:

performance-calibration-topRanked-onsetfixed-0-typedecline-2-flag1-1-method-0-dist-0-daily-coronavirus-cases-USA-area-52-05-11-2020.csv

4) Doubling times for each of the top-ranked models:

doublingTimes-**ranked(1)**-onsetfixed-0-typedecline-2-flag1-1-method-0-dist-0-daily-coronavirus-cases-USA-area-52-05-11-2020.csv

doublingTimes-**ranked(2)**-onsetfixed-0-typedecline-2-flag1-1-method-0-dist-0-daily-coronavirus-cases-USA-area-52-05-11-2020.csv

doublingTimes-**ranked(3)**-onsetfixed-0-typedecline-2-flag1-1-method-0-dist-0-daily-coronavirus-cases-USA-area-52-05-11-2020.csv

doublingTimes-**ranked(4)**-onsetfixed-0-typedecline-2-flag1-1-method-0-dist-0-daily-coronavirus-cases-USA-area-52-05-11-2020.csv

A relevant issue to investigate when using any mathematical model is that of structural or practical parameter identifiability [43]. Structural identifiability arises when one or more model parameters cannot be uniquely estimated using the model, even when the data is free of noise. That is, a lack of structural identifiability is due to issues in the model structure, such as the presence of parameter correlations [12]. On the other hand, practical identifiability occurs when one or more parameters cannot be reliably estimated using the available observed data, which could be associated with the number of observations available for model calibration and the spatial-temporal resolution of the data. Because the time series of incident cases in the observed epidemic wave is an aggregation of overlapping sub-epidemics, there could be instances when different sub-epidemic profiles may give rise to indistinguishable aggregated epidemic waves as noted elsewhere [44].

### Generate the top-ranked and ensemble sub-epidemic model forecasts and the associated forecasting performance metrics

Using the function plotForecast_SW_subepidemicFramework.m, we can plot the short-term forecasts from the top-ranking sub-epidemic models and the ensemble models derived from the top-ranking sub-epidemic models based on the inputs indicated in the options.m and the options_forecast.m files. However, this function can also receive parameters < outbreakx>, <caddate1>, or <forecastingperiod> as passing input parameters while the remaining inputs are read from the options.m and options_forecast.m files. Moreover, the data associated with each top-ranked model and ensemble forecasts are saved as .csv files in the output folder.

In addition, this function also plots the forecasting performance metrics (MSE, MAE, 95% P.I., WIS) for the top-ranking models and the ensemble sub-epidemic wave models. Finally, this function also stores *.csv files in the output folder with the forecasting performance metrics associated with the top-ranking and ensemble models, and the estimated doubling times for each of the top-ranked models. Using the default parameter values indicated in the options.m, and options_forecast.m files, the call to this function from MATLAB’s command line follows:

>> **plotForecast_subepidemicFramework**

**Fig. 14:**
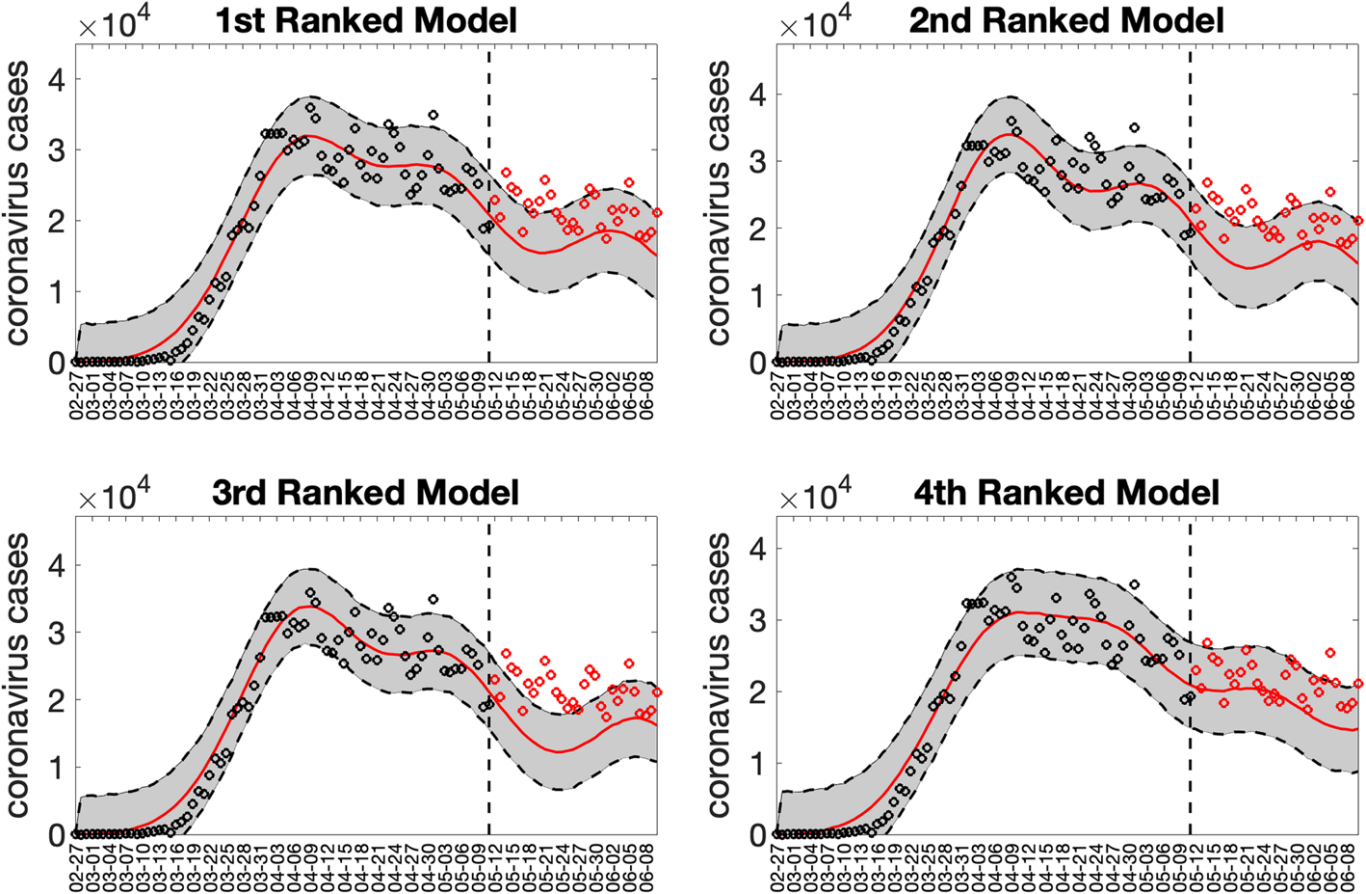
30-day forecasts derived from the top-ranking sub-epidemic models fit to the daily curve of COVID-19 cases in the USA from 11-May-2020 to 10-June-2020. The model fit (solid line) and 95% prediction interval (shaded area) are also shown. The vertical line indicates the start time of the forecast and separates the calibration and forecast periods. Circles correspond to the data points. Of note, the data associated with each top-ranked model forecast are also saved as .csv files in the output folder

**Fig. 15:**
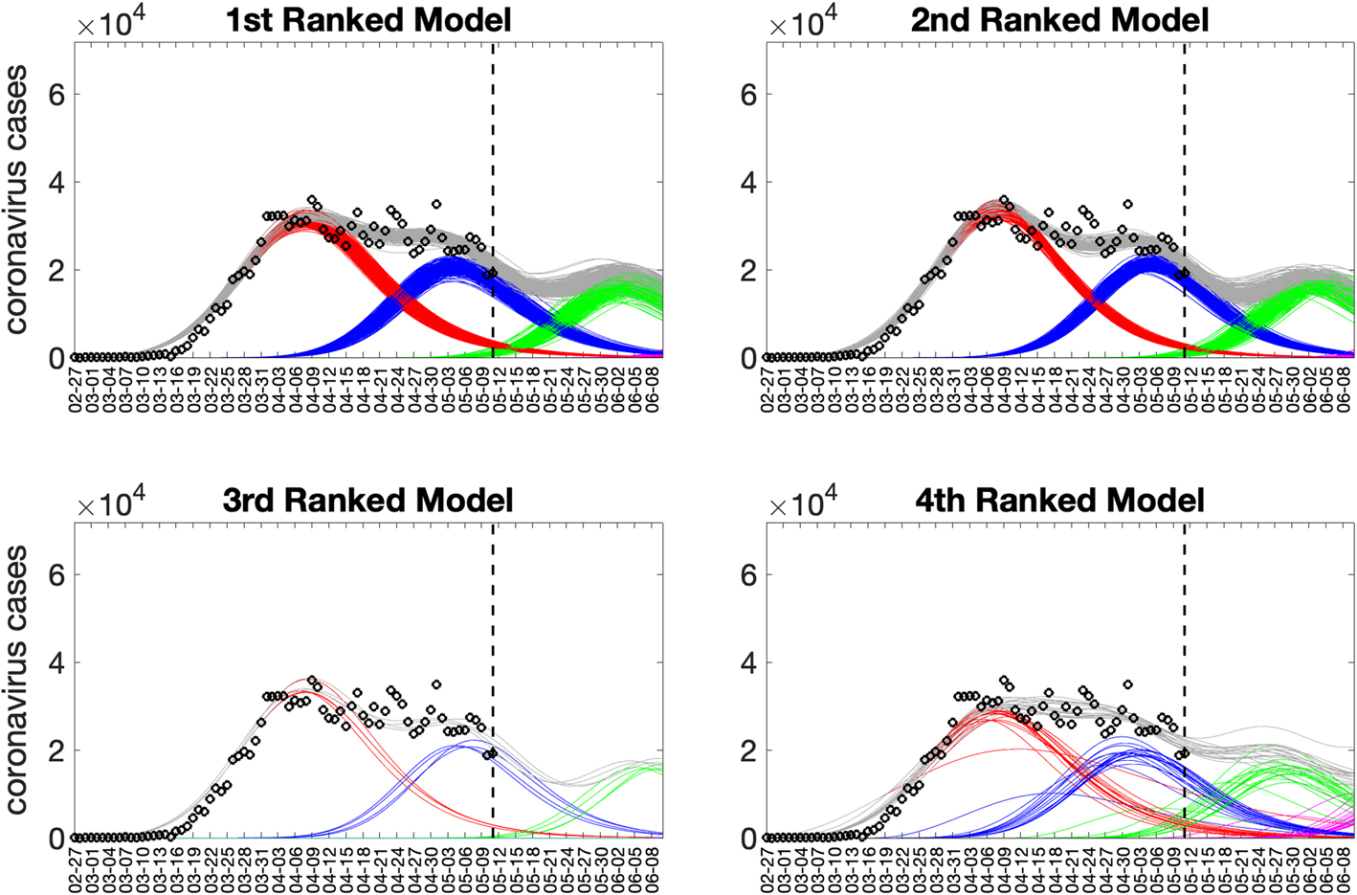
Sub-epidemic profiles of the 30-day forecasts derived from the top-ranking sub-epidemic models fit to the daily curve of COVID-19 cases in the USA from 11-May-2020 to 10-June-2020. The epidemic wave’s sub-epidemic mean curves obtained from the parametric bootstrapping with 300 bootstrap realizations are shown in red, blue, green, and magenta. The gray curves correspond to the overall epidemic trajectory obtained by aggregating the individual sub-epidemic curves. The vertical line indicates the start time of the forecast and separates the calibration and forecast periods

**Fig. 16:**
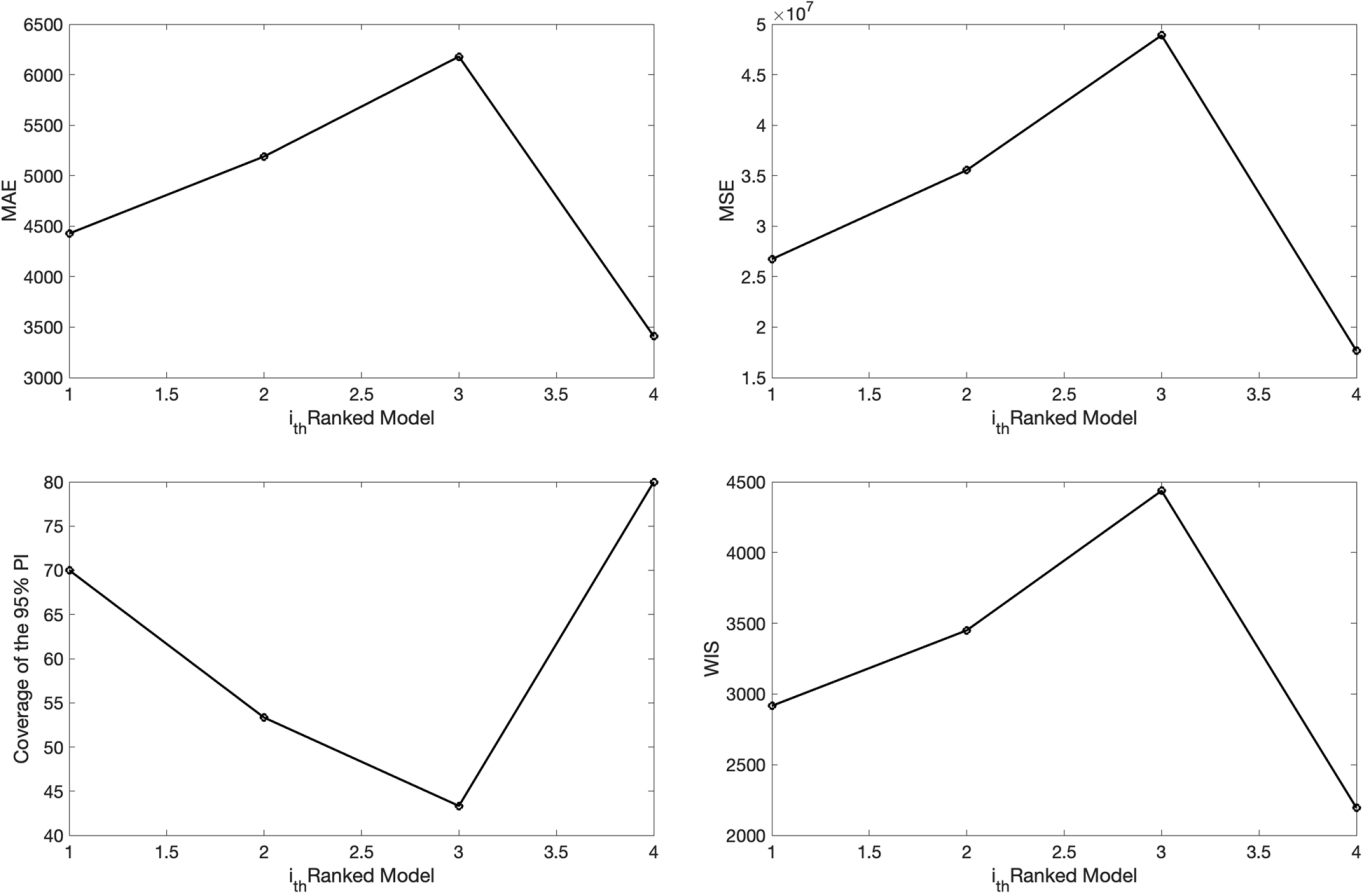
30-day forecasting performance metrics derived from the top-ranking sub-epidemic models for the daily curve of COVID-19 cases in the USA from 11-May-2020 to 11-June-2020. The forecasting performance metrics are also saved in a .csv data file in the output folder (‘performance-forecasting-topRanked-onsetfixed-0-typedecline-2-flag1-1-method-0-dist-0-horizon-30-weight_type-1-daily-coronavirus-cases-USA-area-52-05-11-2020.csv’)

Figures 14 and 15 illustrate the outputs obtained from this function call. Figure 14 shows the 30-day forecasts derived from the top-ranking sub-epidemic models, whereas Fig. 15 shows the sub-epidemic profiles of the forecasts. These forecasts indicate that the 1st-ranked model outperformed the other top-ranked models. Moreover, the data associated with the top-ranked model forecasts are also saved as .csv files in the output folder. The forecasting performance metrics for the top-ranked models are displayed in Fig. 16, and these metrics are also saved in a .csv file in the output folder. In comparison, the forecast derived from the simpler growth model consisting of a single sub-epidemic (<npatches_fixed>=1) was substantially worse, as shown in Supplementary Fig.  2.

**Fig. 17:**
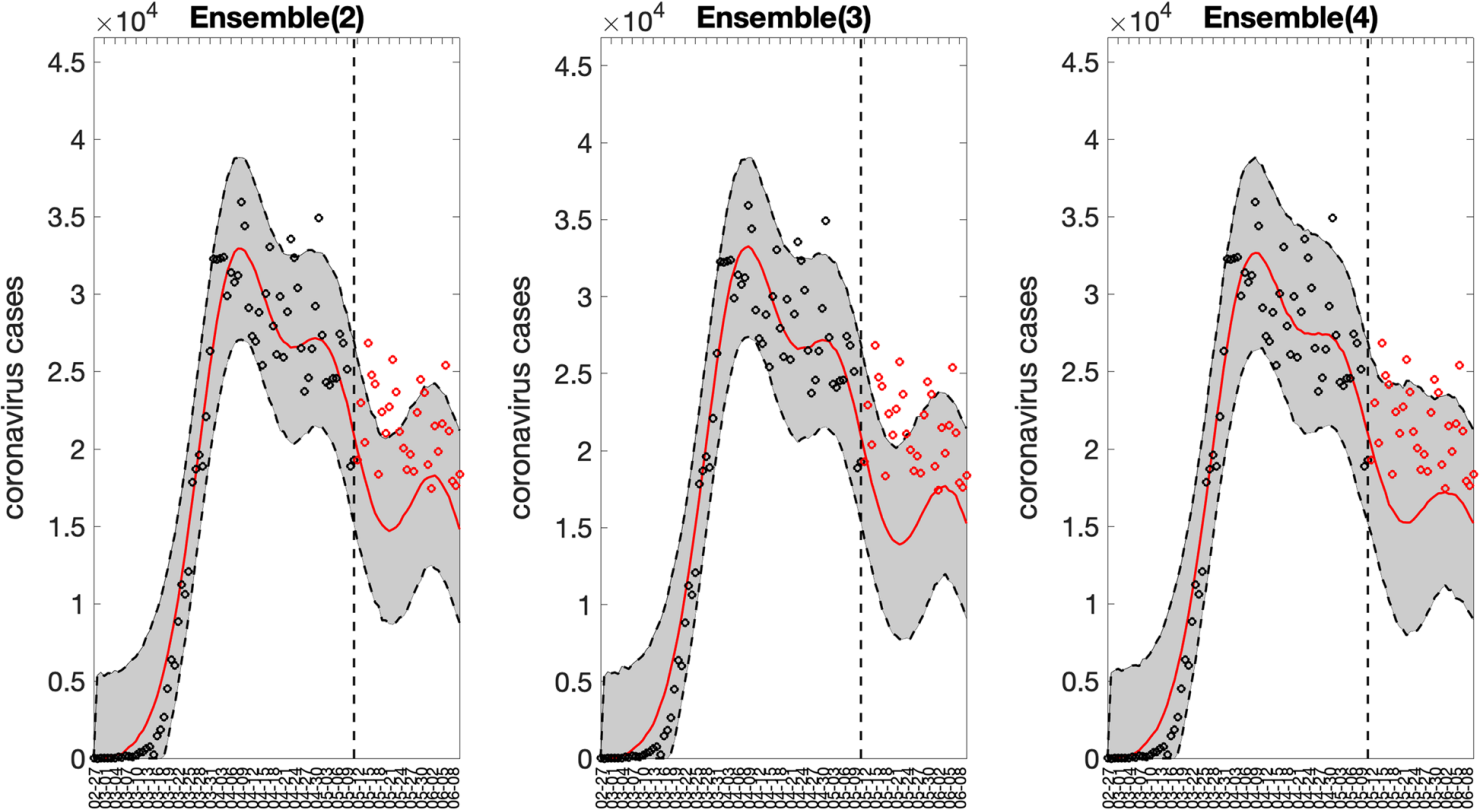
30-day sub-epidemic ensemble model forecasts (Ensemble(2), Ensemble(3), Ensemble(4)) of COVID-19 cases in the USA from 11-May-2020 to 11-June-2020. Circles correspond to the data points. The model fits (solid line), and 95% prediction intervals (shaded area) are shown. The vertical line indicates the start time of the forecast. Of note, the data associated with each ensemble model forecast are also saved as .csv files in the output folder

**Fig. 18:**
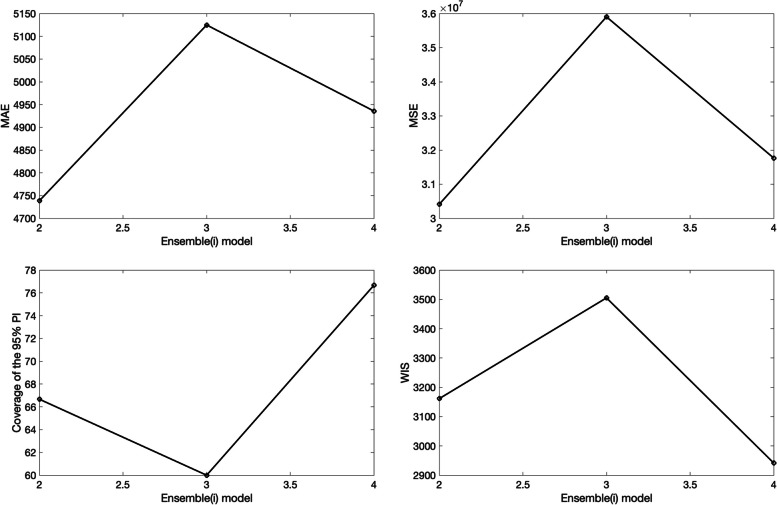
30-day forecasting performance metrics derived from the sub-epidemic ensemble models for the daily curve of COVID-19 cases in the USA from 11-May-2020 to 11-June-2020. The performance metrics are also saved in a .csv data file in the output folder (‘performance-forecasting-Ensemble-onsetfixed-0-typedecline-2-flag1-1-method-0-dist-0-horizon-30-weight_type-1-daily-coronavirus-cases-USA-area-52-05-11-2020.csv’)

The corresponding 3 ensemble forecasts (Ensemble(2), Ensemble(3), and Ensemble(4)) derived from the weighted combination of the top-ranked models based on their relative likelihood or Akaike weights (e.g., < weight_type1>=1 in the options_forecast.m file) are shown in Fig. 17. Also, the corresponding forecasting performance metrics for the ensemble models are shown in Fig. 18 and are saved in a .csv file in the output folder. The Ensemble(4) performed slightly better than the Ensemble(2) and Ensemble(3) models in terms of the WIS and coverage of the 95% prediction interval. This function will store the following .csv files in the output folder:

1. Forecasting performance metrics of the top-ranked models:

performance-forecasting-topRanked-onsetfixed-0-typedecline-2-flag1-1-method-0-dist-0-horizon-30-daily-coronavirus-cases-USA-area-52-05-11-2020.csv

2) Forecasting performance metrics of the ensemble models:

performance-forecasting-Ensemble-onsetfixed-0-typedecline-2-flag1-1-method-0-dist-0-horizon-30-weight_type-1-daily-coronavirus-cases-USA-area-52-05-11-2020.csv

3) Forecasts of the top-ranked models:

**ranked(1)**-onsetfixed-0-typedecline-2-flag1-1-method-0-dist-0-horizon-30-daily-coronavirus-cases-USA-area-52-05-11-2020.csv

**ranked(2)** -onsetfixed-0-typedecline-2-flag1-1-method-0-dist-0-horizon-30-daily-coronavirus-cases-USA-area-52-05-11-2020.csv

**ranked(3)** -onsetfixed-0-typedecline-2-flag1-1-method-0-dist-0-horizon-30-daily-coronavirus-cases-USA-area-52-05-11-2020.csv

**ranked(4)** -onsetfixed-0-typedecline-2-flag1-1-method-0-dist-0-horizon-30-daily-coronavirus-cases-USA-area-52-05-11-2020.csv

4) Forecasts of the ensemble models:

**Ensemble(2)**-onsetfixed-0-typedecline-2-flag1-1-method-0-dist-0-horizon-30-weight_type-1-daily-coronavirus-cases-USA-area-52-05-11-2020.csv

**Ensemble(3)** -onsetfixed-0-typedecline-2-flag1-1-method-0-dist-0-horizon-30-weight_type-1-daily-coronavirus-cases-USA-area-52-05-11-2020.csv

**Ensemble(4)** -onsetfixed-0-typedecline-2-flag1-1-method-0-dist-0-horizon-30-weight_type-1-daily-coronavirus-cases-USA-area-52-05-11-2020.csv

5) Sequence of doubling times of the top-ranked models:

doublingTimes-**ranked(1)**-onsetfixed-0-typedecline-2-flag1-1-method-0-dist-0-horizon-30-daily-coronavirus-cases-USA-area-52-05-11-2020.csv

doublingTimes-**ranked(2)**-onsetfixed-0-typedecline-2-flag1-1-method-0-dist-0-horizon-30-daily-coronavirus-cases-USA-area-52-05-11-2020.csv

doublingTimes-**ranked(3)**-onsetfixed-0-typedecline-2-flag1-1-method-0-dist-0-horizon-30-daily-coronavirus-cases-USA-area-52-05-11-2020.csv

doublingTimes-**ranked(4)**doublingTimes--onsetfixed-0-typedecline-2-flag1-1-method-0-dist-0-horizon-30-daily-coronavirus-cases-USA-area-52-05-11-2020.csv

6) Sequence of doubling times of the ensemble models:

doublingTimes-**Ensemble(2)**-onsetfixed-0-typedecline-2-flag1-1-method-0-dist-0-horizon-30-weight_type-1-daily-coronavirus-cases-USA-area-52-05-11-2020.csv

doublingTimes-**Ensemble(3)**-onsetfixed-0-typedecline-2-flag1-1-method-0-dist-0-horizon-30-weight_type-1-daily-coronavirus-cases-USA-area-52-05-11-2020.csv

doublingTimes-**Ensemble(4)**-onsetfixed-0-typedecline-2-flag1-1-method-0-dist-0-horizon-30-weight_type-1-daily-coronavirus-cases-USA-area-52-05-11-2020.csv

We can also compare the performance of unweighted ensemble models by setting the parameter <weight_type1>=-1 in the options_forecast.m file while the other parameters are kept unchanged. Then we can compare the performance of the unweighted ensemble models (equal weights across top-ranked models) with the weighted ensemble models, where the weights are proportional to the relative likelihood of the models (<weight_type1>= 1). We can run the function plotForecast_subepidemicFramework.m to generate the new set of forecasts with the new models.

The forecasting performance metrics for the weighted and unweighted ensemble models and other statistical time-series models are displayed in Table 2. Overall, the unweighted ensemble models performed similarly as their weighted ensemble counterparts for this forecast and outperformed some popular statistical time-series models such as ARIMA (a brief description of the statistical models is given in Supplementary Text S3).

**Table 2:** Forecasting performance metrics derived from the weighted and unweighted ensemble models, an auto-regressive integrated moving average model (ARIMA), a generalized additive model (GAM), and simple linear regression model (SLR) based on the daily curve of COVID-19 cases in the USA from 11-May-2020 to 11-June-2020. The weights of the weighted ensemble model are based on relative likelihood. Overall, both ensemble types performed similarly for this forecast, and outperformed the simple statistical models

Model	Forecasting period	MAE	MSE	Coverage 95% PI	WIS
**Weighted Ensemble(2)**	30	4716.01	30200654.24	66.67	3156.50
**Unweighted Ensemble(2)**	30	4662.00	29686078.76	66.67	3169.62
**Weighted Ensemble(3)**	30	5229.91	36934107.63	60.00	3490.51
**Unweighted Ensemble(3)**	30	5262.52	37441993.33	60.00	3482.98
**Weighted Ensemble(4)**	30	5023.32	32564946.12	76.67	2926.13
**Unweighted Ensemble(4)**	30	4836.87	30807937.90	76.67	2942.91
**ARIMA**	30	7560.80	77139741.86	90.00	4118.39
**GAM**	30	8345.23	94188590.40	50.00	5466.92
**SLR**	30	23380.65	583817550.48	0.00	21739.18

## Conclusion

We have introduced a MATLAB toolbox to fit and forecast time series using the spatial wave sub-epidemic model originally developed to generate short-term forecasts of epidemics [13] and illustrated its functionality using time-series data of the COVID-19 pandemic in the US. In particular, the sub-epidemic model used in this tutorial has shown competitive performance in characterizing and forecasting epidemic trajectories of infectious diseases such as COVID-19, Ebola, and plague [13, 15]. The toolbox can be a helpful resource for policy makers and used as a part of the curriculum for students training in infectious disease modeling, mathematical biology, applied statistics and mathematics, and special courses in epidemic modeling and time-series forecasting.

This new open-source toolbox and associated tutorial will be helpful to a broad community of applied scientists interested in characterizing and forecasting time-series data that results from the aggregation of multiple asynchronous underlying growth processes. Moreover, prior publications have extensively validated the tools presented here [13, 15]. The models and methods included in the toolbox have improved short-term forecasting performance over simpler growth models such as the Richards and generalized-logistic growth models. Moreover, we have ensured publicly available, long-term, and stable hosting of the toolbox in a public GitHub repository. Extensions to the toolbox could include additional components, such as new model features, alternative estimation methods, and additional forecasting performance metrics.

### Availability and requirements

Project name: Forecasting growth trajectories using the ensemble spatial wave sub-epidemic modeling framework.

Project home page: https://github.com/gchowell/spatial_wave_subepidemic_framework Operating system(s): Platform independent.

Programming language: MATLAB.

Other requirements: NA.

License: This program is free software: it can be redistributed or modified under the GNU Public License as published by the Free Software Foundation, version 3 of the License.

Any restrictions to use by non-academics: None.

### Supplementary Information

Supplementary Material 1.

Supplementary Material 2.

## Data Availability

Datasets for daily COVID-19 cases reported in the USA are retrieved from the publicly available data tracking system of the Johns Hopkins Center for Systems Science and Engineering (CSSE). Code files can be accessed at https://github.com/gchowell/spatial_wave_subepidemic_framework.

## Abbreviations

AIC: Akaike Information Criterion
ARIMA: Auto Regressive Integrated Moving Average
CSSE: Center for System Science and Engineering
CI: Confidence Interval
GLM: Generalized Logistic growth Model
IS: Interval Score
MAE: Mean Absolute Error
MLE: Maximum Likelihood
MSE: Mean Squared Error
ODE: Ordinary Differential Equations
PI: Prediction Interval
USA: United States of America
WIS: Weighted Interval Score

## References

[1] Petropoulos F, Apiletti D, Assimakopoulos V, Babai MZ, Barrow DK, Ben Taieb S, Bergmeir C, Bessa RJ, Bijak J, Boylan JE (2022). Forecasting: theory and practice. Int J Forecast.

[2] Dimri T, Ahmad S, Sharif M (2020). Time series analysis of climate variables using seasonal ARIMA approach. J Earth Syst Sci.

[3] Hyndman RJ, Athanasopoulos G. Forecasting: Principles and Practice. 2nd ed. OTexts. 2018. p. 384.

[4] Mondal P, Shit L, Goswami S (2014). Study of effectiveness of time series modeling (ARIMA) in forecasting stock prices. Int J Sci Eng Appl.

[5] Shamsnia SA, Shahidi N, Liaghat A, Sarraf A, Vahdat SF. Modeling of weather parameters using stochastic methods (ARIMA model)(case study: Abadeh Region, Iran). In: International Conference on Environment and Industrial Innovation. IPCBEE. 2011;12.

[6] Tektaş M (2010). Weather forecasting using ANFIS and ARIMA models. Environ Res Eng Manag.

[7] Yan P, Chowell G (2019). Quantitative methods for investigating infectious disease outbreaks vol. 70.

[8] Chowell G (2017). Fitting dynamic models to epidemic outbreaks with quantified uncertainty: a primer for parameter uncertainty, identifiability, and forecasts. Infect Dis Model.

[9] Chowell G, Castillo-Chavez C, Fenimore PW, Kribs-Zaleta CM, Arriola L, Hyman JM (2004). Model parameters and outbreak control for SARS. Emerg Infect Dis.

[10] Keeling MJ, Hill EM, Gorsich EE, Penman B, Guyver-Fletcher G, Holmes A, Leng T, McKimm H, Tamborrino M, Dyson L (2021). Predictions of COVID-19 dynamics in the UK: short-term forecasting and analysis of potential exit strategies. PLoS Comput Biol.

[11] Viboud C, Sun K, Gaffey R, Ajelli M, Fumanelli L, Merler S, Zhang Q, Chowell G, Simonsen L, Vespignani A (2018). The RAPIDD ebola forecasting challenge: synthesis and lessons learnt. Epidemics.

[12] Tuncer N, Timsina A, Nuno M, Chowell G, Martcheva M (2022). Parameter identifiability and optimal control of an SARS-CoV-2 model early in the pandemic. J Biol Dyn.

[13] Chowell G, Tariq A, Hyman JM (2019). A novel sub-epidemic modeling framework for short-term forecasting epidemic waves. BMC Med.

[14] Raimund B, Gerardo C, Leidy Yissedt L-D (2019). Comparative analysis of phenomenological growth models applied to epidemic outbreaks. Math Biosci Eng.

[15] Chowell G, Rothenberg R, Roosa K, Tariq A, Hyman JM, Luo R (2022). Sub-epidemic model forecasts during the first wave of the COVID-19 pandemic in the USA and European hotspots. Mathematics of Public Health.

[16] Chowell G, Dahal S, Tariq A, Roosa K, Hyman JM, Luo R (2022). An ensemble n-sub-epidemic modeling framework for short-term forecasting epidemic trajectories: application to the COVID-19 pandemic in the USA. PLoS Comput Biol.

[17] Chowell G, Luo R (2021). Ensemble bootstrap methodology for forecasting dynamic growth processes using differential equations: application to epidemic outbreaks. BMC Med Res Methodol.

[18] Banks HT, Hu S, Thompson WC. Modeling and inverse problems in the presence of uncertainty. 1st ed. Chapman and Hall/CRC; 2014. 10.1201/b16760.

[19] Roosa K, Luo R, Chowell G (2019). Comparative assessment of parameter estimation methods in the presence of overdispersion: a simulation study. Math Biosci Eng.

[20] Myung IJ (2003). Tutorial on maximum likelihood estimation. J Math Pyschol.

[21] Friedman J, Hastie T, Tibshirani R (2009). The elements of statistical learning: Data mining, inference, and prediction.

[22] Shanafelt DW, Jones G, Lima M, Perrings C, Chowell G (2018). Forecasting the 2001 foot-and-mouth disease epidemic in the UK. EcoHealth..

[23] Chowell G, Hincapie-Palacio D, Ospina J, Pell B, Tariq A, Dahal S, Moghadas S, Smirnova A, Simonsen L, Viboud C. Using Phenomenological models to characterize transmissibility and Forecast patterns and final Burden of Zika Epidemics. PLoS Curr. 2016;8. 10.1371/currents.outbreaks.f14b2217c902f453d9320a43a35b9583 PMC4922743 27366586

[24] Pell B, Kuang Y, Viboud C, Chowell G (2018). Using phenomenological models for forecasting the 2015 Ebola challenge. Epidemics.

[25] Sugiura N (1978). Further analysts of the data by akaike’ s information criterion and the finite corrections. Commun Stat Theory Methods.

[26] Hurvich CM, Tsai C-L (1989). Regression and time series model selection in small samples. Biometrika.

[27] Gneiting T, Raftery AE (2007). Strictly proper scoring rules, prediction, and estimation. J Am Stat Assoc.

[28] Kuhn M, Johnson K (2013). Applied predictive modeling.

[29] Competitor’s Guide: Prizes and Rules.[https://www.m4.unic.ac.cy/wp-content/uploads/2018/03/M4-Competitors-Guide.pdf].

[30] Tariq A, Chakhaia T, Dahal S, Ewing A, Hua X, Ofori SK, Prince O, Salindri AD, Adeniyi AE, Banda JM (2022). An investigation of spatial-temporal patterns and predictions of the coronavirus 2019 pandemic in Colombia, 2020–2021. PLoS Negl Trop Dis.

[31] Bracher J, Ray EL, Gneiting T, Reich NG (2021). Evaluating epidemic forecasts in an interval format. PLoS Comput Biol.

[32] Hwang E (2022). Prediction intervals of the COVID-19 cases by HAR models with growth rates and vaccination rates in top eight affected countries: bootstrap improvement. Chaos Solitons Fractals.

[33] Roosa K, Tariq A, Yan P, Hyman JM, Chowell G (2020). Multi-model forecasts of the ongoing ebola epidemic in the Democratic Republic of Congo, March 2013-October 2019. J R Soc Interface.

[34] Cramer EY, Ray EL, Lopez VK, Bracher J, Brennen A, Castro Rivadeneira AJ, Gerding A, Gneiting T, House KH, Huang Y (2022). Evaluation of individual and ensemble probabilistic forecasts of COVID-19 mortality in the United States. Proc Natl Acad Sci U S A.

[35] Muniz-Rodriguez K, Chowell G, Cheung CH, Jia D, Lai PY, Lee Y, Liu M, Ofori SK, Roosa KM, Simonsen L (2020). Doubling time of the COVID-19 epidemic by Province, China. Emerg Infect Dis.

[36] Smirnova A, DeCamp L, Chowell G (2021). Mathematical and statistical analysis of doubling times to investigate the early spread of epidemics: application to the COVID-19 pandemic. Mathematics.

[37] Wallinga J, Lipsitch M (2007). How generation intervals shape the relationship between growth rates and reproductive numbers. Proc R Soc B: Biol Sci.

[38] Chowell G, Luo R, Sun K, Roosa K, Tariq A, Viboud C (2020). Real-time forecasting of epidemic trajectories using computational dynamic ensembles. Epidemics.

[39] Ray EL, Reich NG (2018). Prediction of infectious disease epidemics via weighted density ensembles. PLoS Comput Biol.

[40] Burnham KP, Anderson DR (2002). Model selection and multimodel inference: a practical information-theoretic approach.

[41] Hopkins J. CSSE Covid-19 timeseries. GitHub; 2022.

[42] Tariq A. GitHub Repository. 2022.

[43] Roosa K, Chowell G (2019). Assessing parameter identifiability in compartmental dynamic models using a computational approach: application to infectious disease transmission models. Theor Biol Med Model.

[44] Chowell G, Tariq A, Hyman JM (2019). A novel sub-epidemic modeling framework for short-term forecasting epidemic waves. BMC Med.

