# Supplementary material for: SpatialWavePredict: a tutorial-based primer and toolbox for forecasting growth trajectories using the ensemble spatial wave sub-epidemic modeling framework
Source: BMC Med Res Methodol. 2024 Jun 7;24:131. doi: 10.1186/s12874-024-02241-2 (PMC11157887; doi:10.1186/s12874-024-02241-2)
Supplement: Supplementary file 1 — Supplementary Material 1. [file 12874_2024_2241_MOESM1_ESM.docx]

**Supplementary File**

*SpatialWavePredict*: A tutorial-based primer and toolbox for forecasting growth trajectories using the ensemble spatial wave sub-epidemic modeling framework

Gerardo Chowell*^1,2^, Amna Tariq^3^, Sushma Dahal^1^, Amanda Bleichrodt^1^, Ruiyan Luo^1^, James M. Hyman^4^

^1^Department of Population Health Sciences, School of Public Health, Georgia State University, Atlanta, GA, USA

^2^Division of International Epidemiology and Population Studies, Fogarty International Center, National Institutes of Health, Bethesda, MD, USA

^3^Department of Pediatrics, School of Medicine, Stanford University, Palo Alto, CA, USA

^4^Department of Mathematics, Tulane University, New Orleans, LA, USA

*Corresponding author(gchowell@gsu.edu)

**Supplementary Figure 1:** Fit of the simple growth model consisting of 1 sub-epidemic modeled to the daily curve of COVID-19 cases in the USA from 27-Feb-2020 to 11-May-2020. The simple model was unable to capture the entire epidemic period. The best model fit is represented by the solid black line and blue circles correspond to the data points.


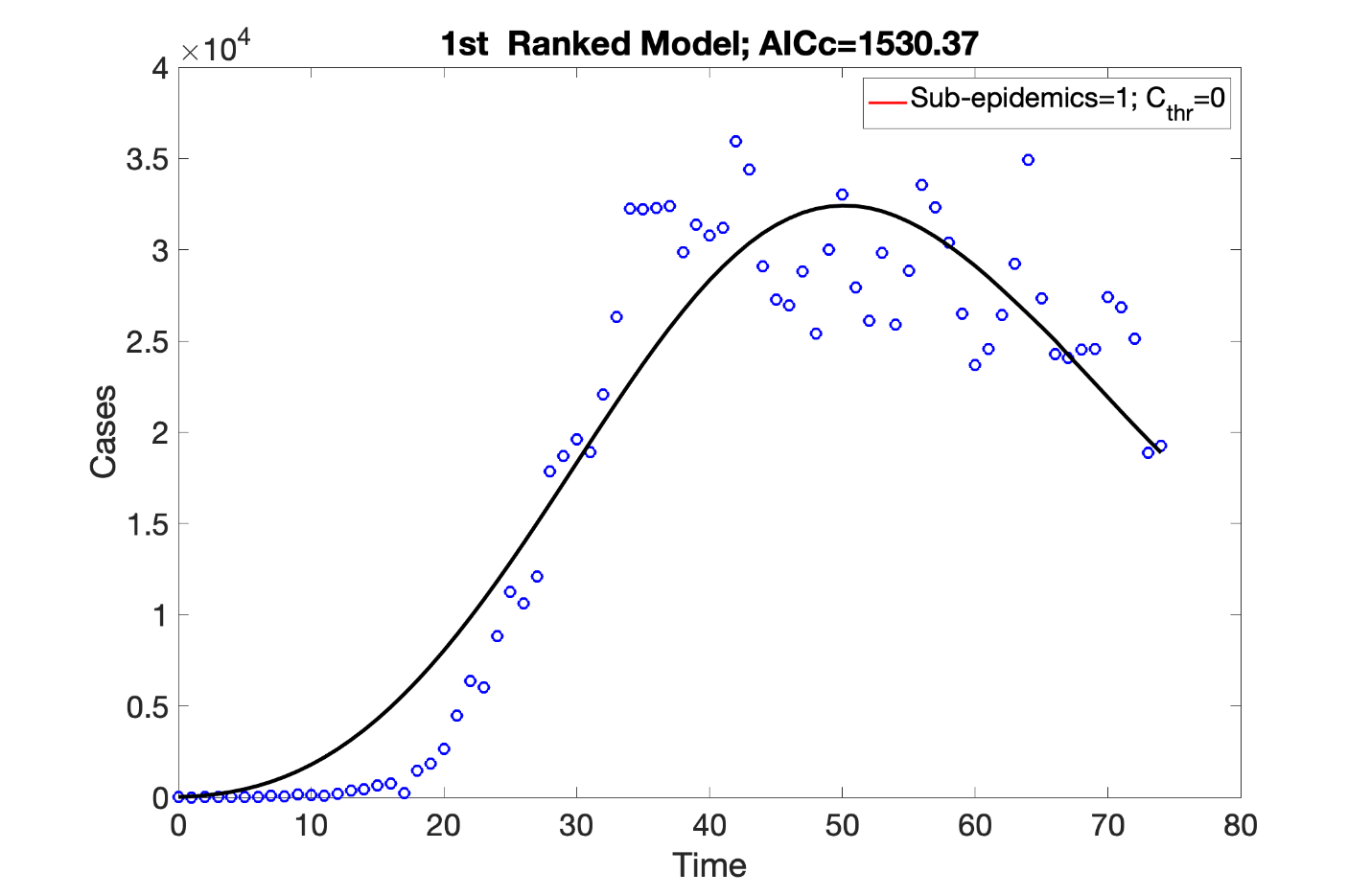


**Supplementary Figure 2:** 30-day forecasts derived from the simple growth model consisting of 1 sub-epidemic modeled to the daily curve of COVID-19 cases in the USA from 11-May-2020 to 10-June-2020. The model fit (solid red line) and 95% prediction interval (shaded area) are also shown. The vertical line indicates the start time of the forecast and separates the calibration and forecast periods. Circles correspond to the data points.


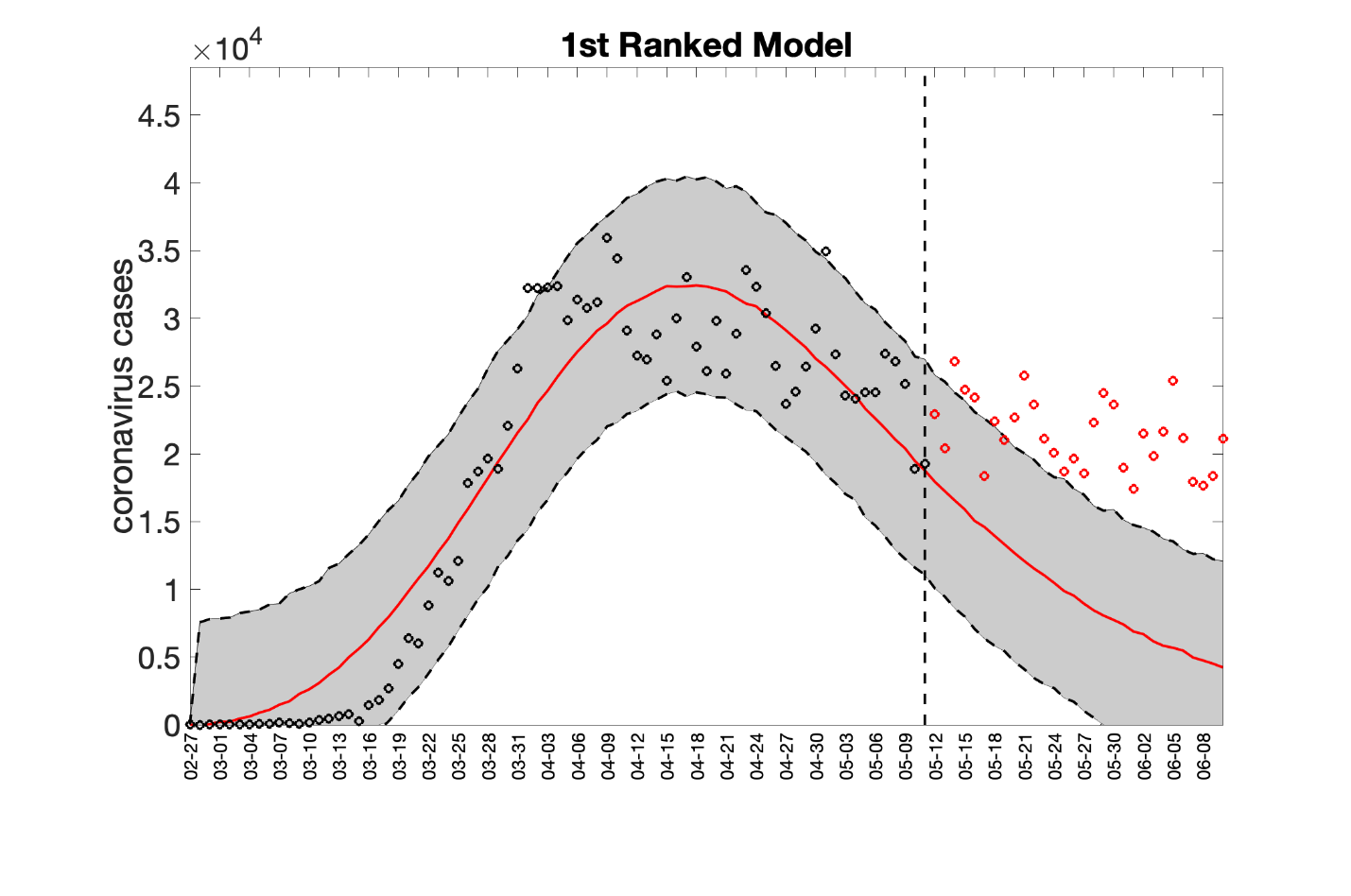


**Supplementary Text 1.**  Structure of the Options.m file for fitting the spatial wave sub-epidemic model to time-series data. The coding blocks define the characteristics of the dataset, adjustments to data, the parameter estimation methods, and the characteristics of the model.

1. **Datasets properties**

This section specifies the characteristics of the time series data, which will be used for inference purposes. The data file is a text file with extension *.txt, located in the input folder.

The data file can contain one or more incidence curves (one per

column in the file). Each column corresponds to the number of new cases over time for each epidemic corresponding to a different area/group. For instance, each column could correspond to different states in the U.S or countries in the world. In the options.m file, a specific data column in the file can be accessed using the parameter <outbreakx> (see below).

if the time series file contains cumulative incidence count data, the name of the time series data file starts with "cumulative" with the following format:

'cumulative-<cadtemporal>-<caddisease>-<datatype>-<cadregion>-<caddate1>.txt');

For example: 'cumulative-daily-coronavirus-deaths-USA-05-11-2020.txt'

Otherwise, if the time series file contains incidence data, the name of the data file follows the format:

<cadtemporal>-<caddisease>-<datatype>-<cadregion>-<caddate1>.txt');

For example: 'daily-coronavirus-deaths-USA-05-11-2020.txt'

The following variables are specified in this section:

<cumulative1>. This is a Boolean variable used to indicate if the data file contains cumulative incidence counts (cumulative1=1) or not (cumulative1=0).

*<outbreakx>*. This is an identifier for the spatial area of interest.

*<caddate1>.* This is a string variable with the data file time stamp in format: mm-dd-yyyy

*<cadregion>.* This is a string variable indicating the geographic region of the time series contained in the file (Georgia, USA, World, Asia, Africa, etc.)

*<caddisease>.* This is a string variable indicating the name of the disease related to the time series data.

*<datatype>.* This is a string variable indicating the nature of the data (e.g., cases, deaths, hospitalizations).

*<DT>.* This variable indicates the temporal resolution in days (1=daily data, 7=weekly data).

*<datevecfirst1>.* This variable contains the date corresponding to the first data point in time series data in format [year_number month_number day_number].

*<datevecend1*>. This is the date of the most recent data file in format [year_number month_number day_number]. This data file is accessed to assess forecast performance.

1. **Adjustments to data**

This section specifies variables relating to smoothing and the calibration period.

The following variables are specified in this section:

*<smoothfactor1>.* This variable indicates the span of the moving average smoothing of the case series (smoothfactor1=1 indicates no smoothing)

*<calibrationperiod1>.* This variable indicates the number of most recent data points that will be used to calibrate the model. If this value exceeds the length of the time series data, it will use the maximum length of the data.

1. **Parameter estimation**

This section specifies the parameter estimation method and the associated assumptions relating to the error structure in the data.

The following variables are specified in this section:

*<method1>.* This integer variable indicates that parameter estimation method employed to estimate the parameters from data. The following estimation methods are available:

method1=0; Nonlinear least squares (LSQ),

method1=1; MLE Poisson=1,

method1=3; MLE (Neg Binomial)=3, with VAR=mean+alpha*mean,

method1=4; MLE (Neg Binomial)=4, with VAR=mean+alpha*mean^2,

method1=5; MLE (Neg Binomial)=5, with VAR=mean+alpha*mean^d.

*<dist1>.* This integer variable indicates the error structure assumptions. The following error structure assumptions are available:

dist1=0; Normal distribution to model error structure (method1=0)

dist1=1; Poisson error structure (method1=0 OR method1=1)

dist1=2; Neg. binomial error structure where var = factor1*mean where

factor1 is empirically estimated from the time series data (method1=0)

dist1=3; MLE (Neg Binomial) with VAR=mean+alpha*mean (method1=3)

dist1=4; MLE (Neg Binomial) with VAR=mean+alpha*mean^2 (method1=4)

dist1=5; MLE (Neg Binomial)with VAR=mean+alpha*mean^d (method1=5)

*<numstartpoints>.* This variable defines the number of different initial guesses for the optimization procedure using Multistart in its search for the globally optimal set of parameters.

*<B>.* Number of bootstrap realizations utilized to characterize parameter uncertainty.

1. **Spatial wave sub-epidemic model**

This section specifies the characteristics of the model.

The following variables are specified in this section:

*<npatches_fixed>.* This variable indicates the maximum number of sub-epidemics considered in the epidemic wave model fit.

*<topmodelsx>.* This variable specifies the number of best fitting models (based on AICc) that will be generated to derive ensemble models. If npatches_fixed=1 then there is only one model.

*<flag1>.* This integer variable specifies the type of growth model used to model a subepidemic.

*<onset_fixed>.* This variable indicates if the onset timing of subepidemics is fixed at time 0 (onset_fixed=1) or not (onset_fixed=0).

*<typedecline2>.* This variable specifies the type of functional declines that will be considered for the sequential sub-epidemic sizes where typedecline2=1 for exponential decline in subepidemic size and typedecline2=2 for power-law decline in sub-epidemic size.

**Supplementary Text S2.** Structure of the Options_forecast.m file for fitting the spatial wave sub-epidemic model to time-series data and generating a forecast. The coding blocks define the parameters relating to the forecast and the weighting scheme employed to derive the ensemble model from the top-ranked models.

**Supplementary Text S3.** Below we provide brief descriptions of the ARIMA and Generalized Additive Model (GAM) methods used in Table 2 for comparison. The simple linear regression (SLR) model in the forecast metrics comparison uses time as the only covariate. As in the main text, all COVID-19 data used in model calibration was smoothed using a seven-day rolling average.

**Auto-Regressive Integrated Moving Average Models (ARIMA)**

The ARIMA(p, d, q) model, commonly employed in disease forecasting [1–5], is given by the following:

$\phi(B)(1-{B)}^{d}y_{t}=c+\theta\left( B \right)\epsilon_{t}.$ (1)

The number of daily COVID-19 cases is represented by $y_{t}$. The backshift operator is given by $B$ implying $By_{t}=y_{t-1}$ and $B\left( By_{t} \right)=B^{2}y_{t}=y_{t-2},$ etc.. This model consists of three parts:

the autoregressive (AR(p)) component $\phi\left( B \right)y_{t}= y_{t}-{\phi_{1}y}_{t-1}-\ldots-{\phi_{p}y}_{t-p}$ about the dependence of $y_{t}$on the p lagged values $y_{t-1},\ldots, y_{t-p}$; the moving average (MA(q)) part $\theta\left( B \right){\epsilon_{t}=\epsilon}_{t}-\theta_{1}\epsilon_{t-1}+\ldots+{\theta_{q}\epsilon}_{t-q}$ about the moving average of errors $\epsilon_{t}, \epsilon_{t-1}, \ldots, \epsilon_{t-q}$; and the differencing part with $(1-B)y_{t}$ representing the difference $y_{t}-y_{t-1}$ and $(1-{B)}^{d}y_{t}$ meaning conducting the differencing $d$ times. . We employed the *auto.arima* function [6] in R to choose the orders p, q, d and build the model, and the *forecast* function of the “forecast” R package [7] was used to produce the evaluated forecast.

**Generalized Additive Models (GAM)**

Like the SLR model, we assumed normality in our GAM implementation and used time as the only covariate. The model is given by [8]:

$y_{t}=\beta_{0}+s\left( t \right)+\epsilon_{t},$ (2)

where $s(.)$ is an unknown smooth function of time, and $\epsilon_{t}\sim N(0, \sigma^{2})$. We use the *gam* function in the “mcgv” package in R [9], where the smooth function $s(.)$ is represented using basis functions or splines:

$s\left( t \right)=\sum_{k=1}^{K} \beta_{k}b_{k}(t).$ (3)

The basis functions are given by $b_{k}(.)$, and $\beta_{k}$ are the expansion coefficients to be estimated [8]. We set the number of basis functions ($K$) to 42. The model was fitted by solving a penalized least squares problem, where a penalty was imposed on the basis coefficients to control the smoothness of $s(.)$. We employed the generalized cross-validation (GCV) criterion option included in the *gam* function in the “mcgv” package to select the smoothness tuning parameter [9]. . A more detailed description of the model fitting methodology can be found in [9], and the associated *predict* function was used for forecasting [10].

**References**

1. Bleichrodt A, Luo R, Kirpich A, Chowell G. Evaluating multi-model and ensemble forecasts in the context of the Mpox outbreak in multiple countries, July 28^th^, 2022, through January 19^th^, 2023. medRxiv. 2023;:2023.05.15.23289989.

2. Chowell G, Dahal S, Tariq A, Roosa K, Hyman JM, Luo R. An ensemble n-sub-epidemic modeling framework for short-term forecasting epidemic trajectories: Application to the COVID-19 pandemic in the USA. PLoS Comput Biol. 2022;18:e1010602.

3. Liu Q, Liu X, Jiang B, Yang W. Forecasting incidence of hemorrhagic fever with renal syndrome in China using ARIMA model. BMC Infect Dis. 2011;11.

4. Aditya Satrio CB, Darmawan W, Nadia BU, Hanafiah N. Time series analysis and forecasting of coronavirus disease in Indonesia using ARIMA model and PROPHET. Procedia Comput Sci. 2021;179:524–32.

5. Chhabra A, Singh SK, Sharma A, Kumar S, Gupta BB, Arya V, et al. Sustainable and intelligent time-series models for epidemic disease forecasting and analysis. Sustainable Technology and Entrepreneurship. 2024;3:100064.

6. Hyndman R. auto.arima: Fit best ARIMA model to univariate time series. RDocumentation. https://www.rdocumentation.org/packages/forecast/versions/8.21.1/topics/auto.arima. Accessed 1 Mar 2024.

7. forecast function. RDocumentation. https://www.rdocumentation.org/packages/forecast/versions/8.4/topics/forecast. Accessed 1 Mar 2024.

8. Shafi A. What are Generalised Additive Models? Towards Data Science. 2021. https://towardsdatascience.com/generalised-additive-models-6dfbedf1350a. Accessed 31 Mar 2023.

9. Wood, S.N. gam: Generalized additive models with integrated smoothness estimation. RDocumentation. https://www.rdocumentation.org/packages/mgcv/versions/1.9-1/topics/gam. Accessed 1 Mar 2024.

10. predict: Model Predictions. RDocumentation. <https://www.rdocumentation.org/packages/stats/versions/3.6.2/topics/predict>. Accessed 1 Mar 2024.

**Supplementary Table 1:** Description of internal functions associated with the toolbox.

| getAICc.m | Computes the AIC_c_ associated with the model fit. |
| --- | --- |
| get_nparams.m | Returns the number of fitting parameters |
| fittingModifiedLogisticFunctionPatchABC.m | Fits a model to the time series data |
| fittingModifiedLogisticFunctionPatchMultiple.m | Generates the uncertainty associated with a given model fit |
| plotModifiedLogisticGrowthPatchMethods1.m | Objective function definition for fitting a model to data |
| modifiedLogisticGrowthPatch.m | Defines the sub-epidemic models for numerical solution |
| AddErrorStructure.m | Generates realizations of the model fit with the specified error structure via parametric bootstrapping. |
| Computeforecastperformance.m | Computes performance metrics for the calibration and forecasting periods (MAE, MSE, coverage of the 95%PI) |
| computeWIS.m | Computes weighted interval score (WIS) to assess model’s performance during the calibration and forecasting periods. |
| getMeanVarianceRatio.m | Computes the average of the variance-to-mean ratios from the time series data to empirically characterize overdispersion using a negative binomial distribution when method1=0 and dist1=2. |
| computeQuantiles.m | Computes the quantiles from the uncertainty of the model separately for the calibration and forecasting periods. |
| getensemblesubepidemics.m | Derives the ensemble forecast from a set of top-ranked sub-epidemic models |
| num2ordinal.m | Returns the ordinal of a number provided as input |
| getData.m | Returns the data needed to evaluate a forecast |
| xticklabel_rotate.m |  |
