# Supplementary material for: SpatialWavePredict: a tutorial-based primer and toolbox for forecasting growth trajectories using the ensemble spatial wave sub-epidemic modeling framework
Source: BMC Med Res Methodol. 2024 Jun 7;24:131. doi: 10.1186/s12874-024-02241-2 (PMC11157887; doi:10.1186/s12874-024-02241-2)
Supplement: Supplementary file 2 — Supplementary Material 2. [file 12874_2024_2241_MOESM2_ESM.zip › Supplementary_video_title_legend.docx]

Supplementary video title: Step by step video tutorial for the manuscript “SpatialWavePredict: A tutorial-based primer and toolbox for forecasting growth trajectories using the ensemble spatial wave sub-epidemic modeling framework”.

Legend: The video illustrates the functionality of the ensemble spatial wave sub-epidemic modeling framework toolbox using the daily COVID-19 case data from the United States of America (USA). The video can be accessed from the following link: https://www.youtube.com/watch?v=qxuF_tTzcR8&t=47s
